# Quality Assessment of Selected Protein Structures Derived from Homology Modeling and AlphaFold

**DOI:** 10.3390/ph16121662

**Published:** 2023-11-29

**Authors:** Furkan Ayberk Binbay, Dhruv Chetanbhai Rathod, Ajay Abisheck Paul George, Diana Imhof

**Affiliations:** 1 Pharmaceutical Biochemistry and Bioanalytics, Pharmaceutical Institute, University of Bonn, An der Immenburg 4, 53121 Bonn, Germany; 2 BioSolveIT GmbH, An der Ziegelei 79, 53757 Sankt Augustin, Germany

**Keywords:** homology modeling, AlphaFold, Gαi1, Gαs, hemopexin, APC, protein model, quality assessment

## Abstract

With technology advancing, many prediction algorithms have been developed to facilitate the modeling of inherently dynamic and flexible macromolecules such as proteins. Improvements in the prediction of protein structures have attracted a great deal of attention due to the advantages they offer, e.g., in drug design. While trusted experimental methods, such as X-ray crystallography, NMR spectroscopy, and electron microscopy, are preferred structure analysis techniques, in silico approaches are also being widely used. Two computational methods, which are on opposite ends of the spectrum with respect to their modus operandi, i.e., homology modeling and AlphaFold, have been established to provide high-quality structures. Here, a comparative study of the quality of structures either predicted by homology modeling or by AlphaFold is presented based on the characteristics determined by experimental studies using structure validation servers to fulfill the purpose. Although AlphaFold is able to predict high-quality structures, high-confidence parts are sometimes observed to be in disagreement with experimental data. On the other hand, while the structures obtained from homology modeling are successful in incorporating all aspects of the experimental structure used as a template, this method may struggle to accurately model a structure in the absence of a suitable template. In general, although both methods produce high-quality models, the criteria by which they are superior to each other are different and thus discussed in detail.

## 1. Introduction

Proteins are highly complex macromolecules that participate in almost all vital biological processes in an organism, including metabolic reactions, transport of molecules, signal transduction, and many more. Great efforts have been made for decades to determine the 3D structures of these essential macromolecules from their primary amino acid sequences, and various methods, including template-based and AI-based methods, have been developed in this context [1]. The key problem therein is that even a small domain of a protein has the potential to fold into an astronomically large number of conformations due to the enormous number of degrees of freedom, particularly of the rotatable bonds of the amino acid side chains [2]. Although it is possible to establish a protein structure with expensive and laborious experimental methods, such as X-ray crystallography, nuclear magnetic resonance (NMR), and cryogenic electron microscopy (Cryo-EM), in silico predictions of protein structures with at least the same quality as experimentally identified equivalents have become an increasingly important goal [3]. Previously, three principal modeling techniques were available, namely homology modeling or comparative modeling, threading (fold recognition), and ab initio modeling [4]. Among these approaches, homology modeling can predict the structure of a protein from a known sequence and experimental structure with a certain degree of homology (>30%) [4]. Bovine α-lactalbumin was the first structure to be predicted by a homology modeling approach in 1969 [5]. Since then, there have been plenty of different applications of this method with many success stories [5,6,7]. However, with the advancement of technologies and the development of different algorithms, homology modeling has gradually lost its reputation to an artificial intelligence (AI)-based method, i.e., AlphaFold (AF) [8,9]. This AI-based method has been recognized as a revolutionary breakthrough in the field of structural biology due to its unprecedented accuracy in predicting unknown structures from an amino acid sequence [9]. Despite the use of high-resolution crystallographic structures in the training of its deep neural network architecture, which also contributes positively to the accuracy of the predicted protein structures [10,11], AF faces several limitations, such as the inability to predict cofactors, metal ions, or bound ligands, although attempts have recently been made to overcome these using methods such as AlphaFill [12].

Herein, the quality of structures predicted by AF and homology modeling techniques is investigated to gain a deeper insight into the prediction qualities. The overall quality of the individual structures as well as the per-residual quality of the structures is assessed. In this study, we focused on seven different human proteins, namely Gαi1 [13], Gαs [13], hemopexin (Hx) [14,15], activated protein C (APC) [16], Rap2 [17], human serum albumin (has) [18], and Interleukin 36α (IL-36α) [19], mainly from our recent studies. Among these proteins, Gαi1, Gαs, and Rap2 are classified as cell membrane-associated proteins. Gαi/s subunits transduce the signal derived from the cell surface receptor GPCR (G protein-coupled receptors) to the effector protein, adenylyl cyclase (AC), inside the cell (Gαi and Gαs act as an inhibitor and a stimulator of AC, respectively) [20]. Like Gαi/s protein subunits, the small GTPase Rap2 participates in different signaling pathways by interacting with and regulating various intracellular effector proteins [17]. The Gαi/s and Rap2 proteins harbor dynamic loop structures surrounding the nucleotide-binding pocket, so-called switch (SW) regions, which are involved in protein activation (Figure S1).

Hx is a glycoprotein with a high heme-binding affinity that is produced in the liver and belongs to the family of acute-phase proteins. It transports heme to its catabolism sites and thus prevents heme-mediated oxidative damage [14]. APC, another heme regulatory protein, is a glycoprotein that is also synthesized in a K-dependent manner in the liver. It has anticoagulant and cytoprotective properties [21]. HSA is the most abundant plasma protein and is known as a carrier and scavenger of different molecules via specific and unspecific binding. HSA scavenges labile heme in the plasma and facilitates its passage to heme degradation pathways [22].

By upregulating the expression of inflammatory and cartilage catabolic markers, IL-36α functions as a pro-inflammatory cytokine at the cartilage level. Reduced p38 activation as well as IL-6 and IL-8 mRNA levels in human fibroblast-like synoviocytes from rheumatoid arthritis patients indicate a considerable reduction in IL-36-mediated signaling upon heme binding [19,23]. The main reason for focusing on these proteins is that first, they contain multiple functional domains that allow us to observe how accurately each domain is predicted in addition and relative to the overall protein structure, and second, they harbor specific binding sites, such as the nucleotide-binding pocket in Gαi/s protein subunits and Rap2 protein or heme-binding motifs in APC, Hx, HSA, and IL-36α. The conformational states of such binding sites are key determinants of function and can often be related to the conformation and behavior of other regions, e.g., allosteric sites, within the protein of interest. Therefore, precise and meticulous modeling of such specific regions of proteins plays a crucial role in determining the accuracy of the computational studies to be carried out. In this context, the quality of the models predicted by both homology modeling and AF were compared with each other and with experimentally resolved structures. In addition, the reliability of these predicted models for utilization in structure-based drug design methods such as molecular docking and structure-based virtual screening [24] was evaluated.

The results here intend to serve as a deep dive into the 3D structure prediction algorithms AlphaFold and homology modeling. On top of that, the pipeline created to analyze these structures can be readily used to determine the quality of the predicted structure from any algorithm. In the end, we suggest steps to increase the quality of the predicted structures with various in silico methods.

## 2. Results

**Figure 1:**
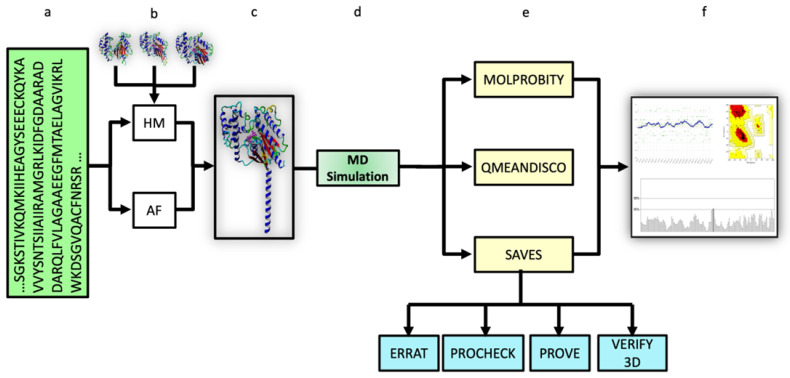
Schematic representation of the workflow for the quality evaluation of computationally predicted protein structures. (**a**) FASTA sequence used as input for modeling, (**b**) computational approaches applied for modeling, (**c**) predicted protein structure, (**d**) molecular dynamics (MD) simulation, (**e**) tools for quality verification of the predicted models, and (**f**) data acquisition for quality control of the predicted models and evaluation.

Once the structures of the proteins of interest (Gαi1, Gαs, APC, Hx, Rap2, IL-36α, and HSA) were generated from the corresponding FASTA sequences (also from the template structures selected for homology modeling), the predicted structures were subjected to a series of systematic probes of their quality by online validation tools. An in-depth analysis of quality metrics such as the accuracy of the folding, the presence of steric clashes between two unpaired atoms, the residue-wise stereochemical quality of the protein structure, and others (Figure 1) was performed. Based on the structural data obtained, protein structures were first analyzed on the basis of the accuracy of prediction of the functional sites, e.g., binding sites, and then the performance of both in silico approaches was evaluated.

### 2.1. Structure Evaluation and Comparison of Homology Models and AlphaFold Structures

The evaluation of the structures of the created homology models (HMs) was performed in situ in YASARA with the overall Z-score, which is a model quality score obtained by averaging the three most precise WHAT IF checks (Ramachandran plot, backbone conformation, and 3D packing quality) provided by YASARA [25]. The Z-score gives information about the extent to which the quality of the model deviates from the average high-resolution crystal structure. A Z-score value greater than zero indicates that the model is optimal, while values less than zero mean that the model deteriorates compared to an average X-ray structure. The Z-scores of the HMs of Gαi1, Gαs, Hx, APC, Rap2, IL-36α, and albumin were found to be 0.67 [13], 0.52, −1.07, −1.41, 0.80, −0.198, and 0.486, respectively, whereas the Z-scores of the AF structures of Gαi1, Gαs, Hx, APC, Rap2, IL-36α and albumin were 0.74, 0.41, −1.16, −1.54, 0.01, −0.58, and 0.43, respectively. Accordingly, Gαi1, Gαs, Rap2, and albumin were classified as optimal, while Hx, APC, and IL-36α were ascertained as satisfactory. Additionally, the predicted local distance difference test (pLDDT) scores, which provide insights into how well the predicted models reconstructed the local atomic interactions in comparison to the pretrained experimental structures [8,11] for the proteins Gαi1, Gαs, and Rap2 revealed that, apart from the overall protein structures, residues in the SW regions (Figure S1), which play an essential role in nucleotide binding, were accurately modeled with high confidence (Table S1). In the AF models of Hx and APC, the aforementioned heme-binding motifs were generally modeled at moderate to high confidence, apart from two motifs from Hx (PGRGH^236^GHRN and RGHGH^238^RNGT) and one motif from APC (TGWGY^391^HSSR), which were modeled at a low confidence level (Table S1). Although the overall quality of the structures was determined by comparison with the corresponding average crystal structures, in addition, structural alignments were also performed between the computationally and the experimentally determined structures to observe how much the modeled structures deviated from the experimental structures (Table 1). The mentioned experimental structures also served as templates for the HMs of the respective proteins (5JS8 [26], 3UMS [27], and 1Y3A [28] for Gαi1; 6EG8 [29], 7E5E [30], and 6AU6 [31] for Gαs; 1QJS [15] for Hx; 1AUT [32], 2AER [33], 3F6U [34], 1W0Y [35], and 3HPT [36] for APC; 2RAP [37] and 3RAP [17] for Rap2; 1AO6 [18], 1N5U [38], and 4G03 (https://www.rcsb.org/structure/4G03, accessed on 6 November 2023) for HAS; and 6HPI [39] for IL-36α). The structural alignments of each experimental structure with the homology and AF models were conducted using MUSTANG [40], which is a multiple structural alignment algorithm in YASARA. By using this method, in addition to observing how much the structures deviate, the sequence identities between the experimental and predicted structures were calculated.

As a result of the structural alignment of the HMs and AF structures with the respective crystal and NMR structures, it was observed that the structures generated by both computational methods are not much different from the experimental structures (Figures S2 and S3). In addition, HMs and AF structures of the same protein were also subjected to structural alignment to examine their similarities. Negligible deviations of 1.17 Å for Gαi1, 0.99 Å for Gαs, 0.98 Å for Hx, and 0.92 Å for APC between the HMs and structures predicted by AF were found. In this context, it was also noted that the structures created in both computational ways are not distinct from each other, although if the numeric RMSD values are used as a strict metric to pick a winner in each case, AF-derived structures have the advantage. The general folding and organization of protein structures were checked to detect deviated residues and to inspect the condition of functionally important regions.

### 2.2. Evaluation of the Gαi1 Structural Models

**Figure 2:**
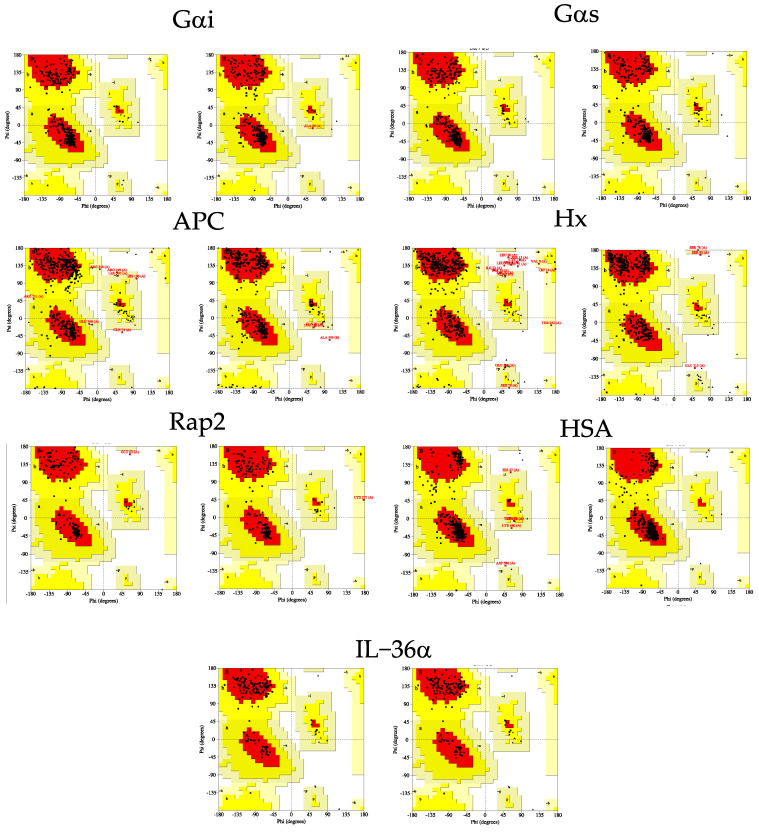
Ramachandran plots of computationally generated protein models. The plots on the left of the heading are from the homology models while the plots on the right-hand side are from the AlphaFold models. The residues found in either generously allowed or disallowed regions are shown in red.

When the steric clashes between two non-bonded atoms in the modeled structures were evaluated on the basis of clashscore, it was observed that the Gαi1 HM does not contain any steric clashes (owing to the energy minimization procedure performed as the final step of the homology modeling workflow in YASARA) (Table 2), while the AF-derived structure showed minimal steric clashes, but less than the other template structures except 5JS8 [26]. Whereas no unfavorable rotamer is present in the AF structure, two residues (Val185 and Glu239) were observed to have unfavorable rotamers (0.67%) in the HM [26]. The HM structure, however, harbors fewer poor side chain rotamers compared to the experimentally determined structures, especially 1Y3A [28] and 5JS8 [26]. Unfavorable rotamers in the HM can be expected due to the presence of poor rotamers in the experimental structures used as templates (Table 2). The absence of Ramachandran outliers in both models indicates that the backbone torsional angles of all residues were indeed favorably constructed (Figure 2). The Rama-Z scores of −0.65 and 0.06 for the homology and AF models, respectively, suggest that the backbone geometry of both models was optimal, (between −2 and 2 [41]), but that of the structure predicted by AF was more regular. Although the Z-score calculated by YASARA also takes into account the torsion angle parameter while providing an overall quality score, the Rama-Z score, which provides insight into the accuracy of the torsion angle distribution [41], was used as a detailed metric to assess the quality of the backbone geometry of the predicted models. The Ramachandran plot revealed that the backbone ϕ/ψ torsional angles were accurately constructed for both homology and AF models since almost all of the residues in both models were in the favored region and no residue was detected in the disallowed region (Figure 2). In the HM, only the residue Ala59 located in the linker I region, a short loop connecting two domains, was found to belong to the generously allowed region (Figure 2). Obtaining positive G-factors, a log-odds score of stereochemical parameters such as torsional angle and covalent geometry, for both models indicate that the overall stereochemistry of both models was indeed optimal.

The mean 3D/1D scores of the residues in the homology and AF models were determined as 91.69% and 85.31%, respectively. Although this showed that the 3D structure of both models was compatible with the primary amino acid sequences, and thus validated the models, it is clear that HM performed better in terms of compatibility. In the HM, 29 residues with a poor compatibility score (<0.2) were found (Figure S3a), five of which (Thr177–Thr181) are located in the SWI region. On the other hand, it was observed that 52 residues scored less than 0.2 in the AF model (Figure S3b*)*. All residues constituting the SWI region (Val174–Gly183), as well as some residues in the SWIII region (Ala235–Glu239), were observed to have low compatibility with their sequences. The overall quality factor based on the non-bonded atomic interactions of both models (99.41% for HM and 98.26% for the AF structure) predicted that both models are of high quality (>95%). In general, nearly all of the residues in the HM (with the exception of two residues) have error rates below the specified threshold of 95% (Figure S4a). However, in the AF model, while five residues were found to have an error rate between 90 and 95%, and a critical error (>95%) was observed in one residue (Leu268) (Figure S4b). But overall, both models have the characteristics of high quality.

The atomic volume Z-scores of 0.89 and 1.16 for the HM and AF structures, respectively, indicate that the volumes of all atoms in the HM are more regular. However, the extremely high standard deviation for both models (Table 2) indicates that the number of outliers in both proteins is excessive. In addition, the astronomical RMSD of the volume Z-score (Z-score rms for both models) gives information about how irregular the buried atoms in both models are [42].

**Figure 3:**
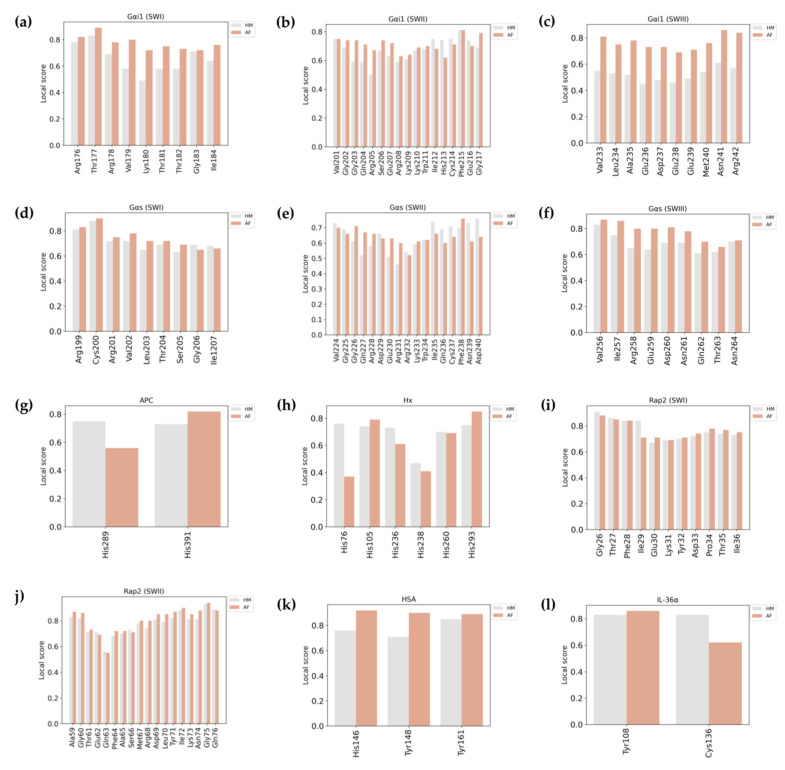
QMEANDisCo scores of the functional residues in the HM and AF-predicted models. Bars representing the functionally important residues in the HM and AF models are shown in grey and coral, respectively. Local QMEANDisCo scores of (**a**–**c**) residues in SWI-III of Gαi1, (**d**–**f**) residues in SWI-III of Gαs, (**g**) coordinating residues in APC, (**h**) coordinating residues in Hx, (**i**,**j**) residues in SWI-II of Rap2, (**k**) coordinating residues in HSA, and (**l**) coordinating residues in IL-36α are illustrated.

The overall QMEANDisCo scores of the Gαi1 structures are 0.76 for the HM and 0.80 for the AF structure, suggesting that the residues in the AF model are slightly better modeled than those in the HM in terms of the expected pairwise Cα-Cα distances. Four residues in both SWI (Val179–Thr182) (Figure 3a) and SWII (Gly203–Arg205, Arg208) (Figure 3b) of the HM were observed to be modeled in low quality, plus almost all of the residues in the SWIII region (Val233–Met240, Arg242) were identified to be of low quality (Figure 3c). On the other hand, it was observed that all residues in the SWI, II, and III regions in the AF structure were well modeled (Figure 3a–c). Although the overall score of both models was acceptable, it was observed that the residues involved in the nucleotide binding were better modeled in the AF model than those in the HM.

### 2.3. Evaluation of the Gαs Structural Models

In the Gαs HM, there is no steric clash between any two non-bonded atoms, whereas a low level of steric clash was found in the AF structure. With this result, the HM again outperformed all template structures by having the lowest steric clash, since it was automatically subjected to energy minimization immediately following its generation (Table 3). Five residues with unfavorable rotamers were found in the HM, while no poorly modeled rotamer was present in the AF structure. It was observed that the number of favored rotamers of both the HM and AF structure was within the defined optimal level (>98%). Additionally, Ramachandran outliers were not detected in any of the computationally determined structures of Gαs. The fact that the Rama-Z scores of both structures were both at the specified level and close to zero indicates that the models have a desirable structure. Moreover, both models appeared to have favorable backbone torsion angles and an optimally constructed backbone (Figure 2).

Both models were noted to possess almost identical Ramachandran analysis results. No residue was found in either allowed or disallowed regions (Table 3). The proportion of residues belonging to the most favored regions is higher than the specified threshold of 90% for both models. Possessing positive G-factors, on the other hand, points out that both models have favorable stereochemical properties. The average 3D/1D scores of the residues were identified as 87.66% for the HM and 87.82% for the AF structure, meaning that the overall compatibility of the residues with their 3D structure is optimal. A total of 47 residues with poor compatibility appeared in the HM, including all residues in the SWI region (Cys187–Gly193) (Figure S5a). On the other hand, 48 residues in the Gαs AF structure, only one of which is in the SWI region, were observed to have low compatibility (Figure S5b). In both models, the residues in the other SW regions were found to be concordant with the 3D models. 

The ERRAT overall quality scores of 99.73% for HM and 98.67% for AF structure prove that both models are of high quality in terms of the non-bonded atomic interactions. Although only one residue had an error rate between 95% and 99% in the HM, four residues in the AF structure were found to have an error rate of 95% to 99%, and one residue had an error rate of slightly more than 99% (Figure S6). However, both models had the properties of a model with a high-quality resolution.

For both protein models, the volumes of the atoms were appropriately formed within the standard atomic volumes of the atoms (Table 3). Moreover, their Z-score RMSD values show that the standard deviations of the atoms in the models are generally within normal ranges except for a few outliers.

The overall QMEANDisCo scores of the HM and AF structure of Gαs were determined as 0.75 and 0.77, respectively. When the SW regions in the Gαs protein were examined, it was observed that every residue in the SWI and SWIII of both models was modeled with ideal quality (Figure 3d–f). Six residues (Gln214, Arg215, and Glu217–Lys220) in the SWII region of the HM of Gαs were observed to have a score of less than 0.60, whereas only one residue located in the SWII region (Arg232) has a score lower than the defined score in the AF model (Figure 3e). In general, the residues in the other SW regions were accurately formed in both models.

### 2.4. Evaluation of the APC Structural Models

Despite the fact that the template structure used for homology modeling consisted of many steric clashes, the model generated from homology modeling consisted of none while the AF model had very minimal clashes. (Table 4). In contrast, only 1.24% of residues were identified as having a poor rotamer for the AF model, while it was 2.28% for the HM model. Even though three out of five templates that were used for generating the HM have below-par favored rotamer percentages, the HM model exhibited a favored region percentage within just below the optimal value (94.59%). The AF structure had the best favored region percentage of 95.02%. Although the Rama-Z scores are at the optimal level for both structures, the high percentage of outliers in the AF model is a reason for caution regarding the overall model quality and backbone structure (Table 4). According to the Ramachandran plot analysis, homology modeling produced a better model as compared to the AF model. There were fewer residues in generously allowed regions and disallowed regions of the HM model than in the AF model. The positive overall G-factor of 0.08 supports this fact in addition to having favorable stereochemical properties (Table 4). When the overall compatibility of the residues with their 3D structures was determined, 28% of the residues of the AF model were incompatible with the 3D structure while in the HM model, only 60.25% of the residues were compatible with the 3D structure, hence it is labeled as a failed model according to this test. (Table 4). The heme-coordinating residues Tyr239 and His391 showed above zero average scores while Tyr239 had a raw score of −0.43 in the HM model (Figure S7a,b). The AF model had the optimum average and raw score (Figure S7c).

The overall quality ERRAT scores were 94.34% and 95.81% for HM and AF models, respectively, representing good model quality in both cases. In the per-residue analysis of ERRAT plots, residues above the 95% limit were almost double in the AF model as compared to HM (Table 4). Additionally, the AF model had four residues over the 99% limit, which is very critical for the overall protein structure quality (Figure S8).

The atomic volume analysis reveals that the templates used for the HM have a very high deviation ranging from 20 to 55 from the standard values. This is reflected in the final HM with the Z-score rms being 26.88, whereas AF has a very favorable Z-score rms of just 1.38, which reflects minor deviations from the standard atomic volume values (Table 4). The overall QMEANDisCo scores of 0.74 and 0.67 for the HM and AF models, respectively, show good quality models globally and are well above the minimum value of 0.6.

As we know, APC is a proven heme-binding protein, and correct prediction of heme binding sites is of prime importance for many types of studies. There are two heme-binding motifs present in the APC, where heme binds to Tyr289 and His391 [16]. When the local QMEANDisCo scores of coordination heme-binding residues were studied, the AF model showed scores of 0.56 and 0.82, while the HM model showed scores of 0.73 and 0.75, respectively (Figure 3g). Hence, one residue of the HBM was predicted with very bad quality by the AF model whereas HM predicted heme-coordinating residues of both the motifs above the minimum score value.

### 2.5. Evaluation of the Hemopexin Structural Models

Non-bonding atomic partners were evaluated for possible steric clash via clashscore analysis. Both HMs and AF models showed the presence of none to minimal steric clashes, respectively (Table 5). There are 0.84% (three residues) poor rotamers present in the HM model, while the value is 1.56% (six residues) for the AF structure. This directly translates to high favored rotamers for the HM model (97.21%) as compared to the AF model (95.05%). A differentiation can be made in the model quality by comparing the Ramachandran outliers. Even though both models have scores above the limit, the HM model has fewer (0.24%) outliers as compared to the very high number (4.13%) present in the AF-derived structure. A lower Ramachandran Z-score of 0.69 for the HM as compared to the higher score of 1.74 for the AF model supports this observation. 

On further analysis based on the Ramachandran plot, it was revealed that the HM model is of better quality than the AF structure as it has higher favored regions (90.3%) as compared to its counterpart (83.6%). Additionally, the AF model (2.1%) has a higher number of residues in generously allowed and disallowed regions than the HM model (0.3%). The HM model’s total G-factor score is positive (0.07), while the AF model’s score is negative (−0.20) as a result. The proof of a high-quality model from HM was further supported by a 95.77% 3D/1D profile score, which means all the residues of the HM model are compatible with its 3D structure whereas only 90% of residues are compatible in the 3D model generated by AF (Figure S9). These high-quality models from HM had no average score below 0.2 (Figure S9a) while the AF model had 29 residues below 0.2 (Figure S9b). These 29 residues do not include any residues from the heme-binding motifs predicted earlier [15].

The HM model exhibited a lower ERRAT score as compared to the AF model (Table 5). There was no significant difference in the residue-wise plots as both showed few regions above the 95% and 99% limits (Figure S10). Another parameter that tips the scale in favor of the better-quality AF model is atomic volume compared with the standard volumes of high-quality structures by PROVE. Even though the template used for the homology modeling had a smaller Z-score (0.49 ± 1.31) and Z-score rms (1.40), the final HM model had a Z-score of (1.09 ± 24.39) and Z-score rms of 24.41, which is very much higher than its counterpart AF structure (1.44) (Table 5).

The overall QMEANDisCo score of the HM model is higher than that of the AF model (Table 5). Both show very good global model quality. Similarly, like APC, hemopexin is also a proven heme binder and correct prediction of HBMs is important. There are six heme coordination residues according to [15]. They are His79, His105, His236, His238, His260, and His293. When the per-residue local QMEANDisCo score was averaged, both the HM and AF models showed very similar scores of 0.66 and 0.62, respectively. Moreover, the individual QMEANDisCo score (Figure 3h) did not show any significant pattern supporting one algorithm over the other. This means that heme-binding motifs were just above the optimum range and could be considered to be moderately predicted by both the prediction technologies.

### 2.6. Evaluation of the Rap2 Structural Models

As with other proteins, no steric clash was found in the HM of Rap2, while minimal steric clash was observed in the AF-predicted model. This suggests that HM exceeds both AF and experimental structures on a protein basis (Table 6). Ser11 in Rap2 HM was found to have an unsatisfactory rotamer. In contrast, no residue with a poor rotamer was observed in the AF structure. Overall, residues in the HM were found to have a sufficient number of favored rotamers (98.09%), while the AF structure had a near-limit number of residues (97.53%). Moreover, no Ramachandran outliers were detected in Rap2 HM; however, Cys177 in AF was observed to have an unfavorable torsional angle. Although the molecular geometries of both predicted models are convenient, it is evident that the backbone of AF is constructed slightly more properly (Figure 2).

Based on the Ramachandran plot, one can say that the ϕ/ψ torsion angles of both predicted models are moderately well generated as the majority of the residues (>90%) are in the favored region. However, Glu62 in HM was noted to be located in the disallowed region in the Ramachandran plot (Figure 2). Furthermore, some residues in HM (10) and AF (15) were found to be in the additional allowed region, and one residue in AF was also found to be in the generously allowed region (Table 6). Although some of the residues in both structures are partially disordered in terms of torsional angle, the fact that both structures have positive G-factors indicates that the backbone stereochemistry of the structures is generally in order. The 3D/1D compatibility scores of 58.19 and 47.54 for the HM and AF-predicted models, respectively, indicate that the folding of amino acid sequences into 3D structures is relatively weak. In both predicted models, feeble compatibility was noted for all residues in SWI (Gly26-Ile36) and some residues in SWII (Ala59-Met67 and Tyr71 in HM; Ala59-Ser66) in AF (Figure S11). 

According to the high ERRAT scores (Figure S12), both methods (95.65% for HM and 98.16% for AF structure) seem to work well on the basis of non-bonded interactions between atoms. Five residues in HM (Ile36, Pro50, Ser51, Leu53, and Gln183) and two residues in AF (Lys42 and Lys172) were found to have an error rate between 95% and 99%. Moreover, it was also observed that residues Glu54 and Ile55 in HM, and Asp173 in AF have error values greater than 99%. However, apart from these erroneous residues, both models show the characteristic features of proteins with high resolution. 

The overall QMEANDisCo score for both structures was 0.83, reflecting that the residues were predominantly modeled with high accuracy. When the SW regions are observed specifically, it is evident that all the residues in the SWI of the predicted models have a confidence score of more than 60% (Figure 3i,j). However, it was noted that both methods modeled the Gln63 residue in the SWII region with a low confidence score (0.56 in HM and 0.55 in AF). Principally, besides the residuals in the SW regions, the two methods seem to generate the residues in the structures with high precision.

### 2.7. Evaluation of the Structural Models of Human Serum Albumin

Non-bonded atomic partners underwent a clashscore analysis to check for any potential steric clashes. The HM had a significantly low clashscore of 0.21 as compared to AF despite the high clashscores of the templates used (Table 7). By contrast, AF had a lower percentage of poor rotamers and Ramachandran outliers as compared to HM and a higher favored rotamer percentage than the AF models. This translated to the lower Rama-Z score of 0.41 as compared to 0.74 given by HM (Table 7). 

The Ramachandran plot analysis revealed that the AF model is slightly better as it has 1% higher favored regions in contrast to HM, which has 93.9% favored regions. Moreover, the AF model had lower additionally and generously allowed regions than the HM model with values of 5.2 and 0.7, respectively. All these positive and negative effects on the structure are reflected in the AF having a 0.24 overall G-factor while HM had a slightly higher G-factor of 0.33. A contrasting result was obtained by Verify3D as the 3D/1D profile score of the AF model was 72.41, which is much lower than its counterpart’s score of 79.12 (Figure S13). The ERRAT results were in line with the 3D/1D profile scores as the AF model scored lower (97.63) than the HM model (98.29) (Figure S14, Table 7). There were five residues above the critical 99% error limit in the AF model whereas it was just two in the HM model.

The overall QMEANDisCo scores of the HM and AF models were 0.81 and 0.84, respectively (Table 7). This signifies the overall good quality of the predicted models. Barring the few terminals’ amino acids that scored lower than the benchmark of 0.6, the individual predictions for all other residues were of very high quality in both approaches.

### 2.8. Evaluation of the IL-36α Structural Models

When the non-bonding atoms were analyzed for the clashscores due to side chain orientation, the differences between the HM and AF models were minor. The AF model had a clashscore of 1.61 while the HM model had a 0 clashscore despite the fact that the template used to generate the model had a very high clashscore of 7.23. The poor rotamer and outlier values of 3 and 1, respectively, for HM models, were nil for the models generated by AF (Table 8). 

Interestingly, the values for Ramachandran most favored regions, additional allowed regions, generously allowed regions, disallowed regions, G-factor, 3D/1D profiles, and ERRAT score were 89.7, 10.3, 0, 0, 0.03, 70.25, and 90.90, respectively (Figures S15 and S16, Table 8). This might occur due to the small size of the protein and only one reference sequence and structure for generating the model. However, the QMEANDisCo scores of the HM were marginally higher than those of the AF (Table 8). Additionally, the HM had better per-residue scores of heme binding regions described [19] than the AF-predicted structures (Figure 3).

### 2.9. Impact of Molecular Dynamics Simulation on Predicted Structures 

Molecular dynamics (MD) simulations of protein structures predicted by both methods were performed to observe how the structures would behave in a dynamic environment as an independent observational approach. The system setup for MD simulation is described in the methodology Section 4.3. 

**Figure 4:**
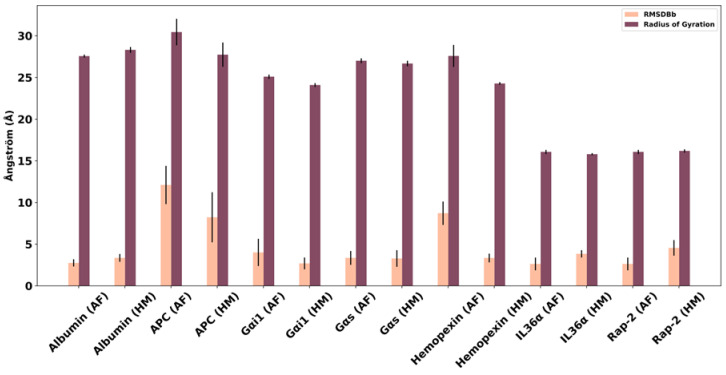
Average backbone RMSD and RadGyration parameters of the predicted structures after MD simulations. The mean RMSD and RadGyration values calculated in the production phase of the HM and AF-predicted structures are shown in peach and claret error bar graphs, respectively. The standard deviation of the values is indicated by the black line.

When the MD simulations of the predicted structures of Gα- proteins were compared with each other, it was observed that Gαs has almost identical RMSDBb (backbone RMSD) and RadGyration (radius of gyration) profiles in both structures (RMSDBb of HM, 3.283 ± 1.001 Å; RMSDBb of AF 3.353 ± 0.827 Å). Additionally, in the simulation trajectories of the structures predicted by both methods, it is seen that the overall folding of the Gαs subunit is the same and hence there is no difference in their compactness as represented by RadGyration (Figure 4). However, in Gαi1, the difference between the mean RMSDBb values of the predicted models is noteworthy. The reason behind this deviation was mainly attributed to the flexibility of the free α-helix located at the N-terminal. The conformational change in the protein structure due to the mobility of the N-terminal helix affected the compactness of the protein as reflected in the RadGyration value. In contrast to Gαi1, Rap2 HM was found to be more deviated compared to its AF counterpart. The principal underlying reason for this is the mobility of the terminal loop, the so-called long wavy hook, which is located at the C-terminus. Moreover, deviations were also observed in the SW regions, which are dynamic loops, of Rap2 HM, compared to AF.

During the simulation of APC, the model generated from HM showed a lower RMSDBb of 8.225 ± 3.0 Å as compared to the AF which had an RMSDBb of 12.102 ± 2.292 Å. The long loop region at the N-terminal is the reason for this higher RMSD change in the AF model (Figure S14). This loop starts folding, thus reducing the RadGyration significantly. As a result, the RadGyration change in the AF model is slightly higher (30.444 ± 1.592 Å) than in the HM model (27.736 ± 1.459 Å). 

In Hx, the AF model had a very high RMSDBb of 8.712 ± 1.401 Å as compared to the HM model which had an RMSDBb of only 3.342 ± 0.518 Å. Similar to APC, a long loop region at the N-terminus contributed to high fluctuations in the dynamic environment (Figure S17). It is also reflected in the RadGyration as a model from AF had to go through high folding fluctuations in contrast to the HM model.

In contrast to APC and Hx, HSA and IL-36α had RMSDBb values of 2.755 ± 0.437 Å and 2.63 ± 0.765 Å from the AF model and 3.352 ± 0.467 Å and 3.843 ± 0.434 Å for the HM model, respectively. The RadGyration also showed much fewer fluctuations for the model predicted by both approaches. The absence of a long loop region might have contributed to this behavior of these two proteins. 

It is interesting to note that the overall quality of the protein was lowered when analyzed for the quality parameters mentioned above (Tables S2–S6). This is often due to factors such as the stereochemical accuracy of the residues in the structure to be used as input to the MD simulation and the precision in modeling their side chains.

## 3. Discussion

The ever-increasing advances in computational approaches to protein modeling have begun to challenge many experimental methods [43]. Fierce competition in this field has yielded a variety of tools for protein prediction, while at the same time experimental studies involving structure analysis using, e.g., cryo-EM are increasing in number, too. In the domain of protein structure prediction, where new computational algorithms often claim to give better results than experimental models, the use of the word artificial intelligence has created hype around the usability of such tools. Especially since the launch of AlphaFold, accuracy in predicting the 3D structure of proteins has reached high levels, with increasing competition between old and newly developed methods for protein structure prediction. In CASP15, it was also observed that with AF version 2, the prediction of protein folding accuracy was further improved with the developments in the underlying neural architecture [44]. In addition, the development of not only AF but also different AI-based methods such as ESMFold [45] and RoseTTAFold [46] are among the factors triggering competition in this field. On the other hand, although experimental determination of the structure of proteins is still one of the most reliable methods, it is likely to be frequently accompanied by AI-based methods, as they are both costly and time-consuming. This project aimed to compare the differences in the quality of the in silico models, as well as the experimentally identified structures. 

Here, we analyzed the precision of the protein structures generated by both AlphaFold and homology modeling and compared them with experimentally determined structures. Both approaches have shown success in modeling Gα- protein subunits and Rap2 protein [13]. The main factor in the evaluation of the Gα- and Rap2 proteins is how accurately the residues in the SW regions involved in nucleotide and effector protein binding are modeled in addition to the overall folding of the individual protein [47]. Specifically, when the organization of residues in the SW regions is examined, it is evident that the AF-predicted model performed better than the HM for the SW regions in the Gαi1 protein subunit, although the homology modeling and AF seem to model the side chains of the Gαs and Rap2 proteins with more or less the same quality. Concerning the heme-binding proteins APC, Hx, HSA, and IL-36α, the analysis results suggest that the homology modeling performs better than AlphaFold when the overall quality of the proteins is considered. The residue-wise analysis of the heme-binding motifs revealed that two motifs from hemopexin and one from APC were poorly predicted by AF. This analysis is relevant as correct modeling of heme-binding sites plays a vital role in predicting transient heme binding to proteins and the further analysis of the structural and functional changes upon heme binding. In addition to this, heme bound to hemopexin was not predicted by AF, while the HM predicted the hemopexin with heme bound to it [14]. 

Although the overall structures of the proteins were predicted with high quality by both models, some minor modeling issues, such as side chain prediction, were observed in both computational approaches; in the generation of the side chains of the proteins, the homology models performed slightly better in modeling the side chains of residues compared to the models predicted with AF. In such cases, performing energy minimization or exposing protein structures to refinement simulations can positively contribute to reducing atomic conflicts, disordered short contacts, and the overall strain, thereby improving the stereochemical accuracy of the residues. The MD simulations conducted on the predicted models proved that AF structures with the long loop regions went through the folding to equilibrate to a stable low-energy conformational ensemble. The capability of AF to predict these loop regions is very low for the proteins whose crystal structure is not used in training the AF algorithm. Additionally, as earlier homology studies were also complemented by MD simulations [14,16], an AF structure should also be subjected to MD simulations before being used for practices such as molecular docking. However, given the fact that the structure used as input may adversely affect the progression of the MD simulation, it is not surprising that the models have different levels of structural deviations. A recent study demonstrated this by subjecting the AF structure to free energy perturbation (FEP) to generate more accurate structures [48]. Overall, it can be said that the local quality of the models predicted by AF is favorable for the application of computer-aided drug design methods.

In the end, we demonstrated the pipeline and parameters through which one can analyze and select the appropriate tool for protein structure prediction without falling for the hype. This approach is valid for the protein structures predicted by any available methods. Knowledge is the real winner here as knowing the limitations of the tool can help a user decide on the method to use; e.g., a user would not select methods like default AF if the protein of interest contains ligands, co-factors, or a loop region to be modeled. On the other hand, if no suitable template is available for comparative modeling, then AI algorithms like AlphaFold come to the rescue. Even though each computational method has advantages and disadvantages compared to the other, the rapid development of AI-based methods is likely to lead to their increasing reputation in this field. However, this should not necessarily mean that homology modeling or experimental methods are gradually becoming obsolete.

## 4. Materials and Methods

### 4.1. Homology Modeling and AlphaFold-Predicted Structures

Homology models (HMs) of Gαi1 [13], Gαs, Hx [14,15], APC [16], Rap2 [17], HSA [18], and IL-36α [19] proteins were generated from experimentally determined structures (Table 9) in YASARA (versions 18.2.7–21.8.27) [15], as described earlier. In general, the structures to be used as templates in this approach were chosen by considering the following criteria: sequence similarity, origin, experimental method used for structural analysis, presence of crucial mutation(s), and how much this would affect the HM to be generated. The existing HMs were used for comparison of the same proteins generated by AF.

The coordinates of all heavy atoms of a protein of interest can be predicted at a high accuracy from the respective amino acid sequence via AF [6]. The prediction of the models can be performed by directly feeding the primary amino acid sequence into a programmatic interface known as AlphaFold Colab [16]. However, the accuracy of the protein structure to be modeled with AF Colab may be decreased due to the potential lack of templates and limited multiple sequence alignment (MSA) resulting from the restricted database (reduced by eightfold) utilized [16]. The AF protein structure database (AF DB, hosted by the European Bioinformatics Institute) covers a wide variety of predicted protein structures, including the human proteome, as well as different other organisms, such as *E. coli* (UniProt ID: UP000000625), *M. musculus* (UniProt ID: UP000000589), and *S. cerevisiae* (UniProt ID: UP000002311) [26]. Therefore, AF DB was queried to check for the presence of the structures of the proteins of interest. Due to the fact that the AF-predicted structures of Gαi1 (UniProt ID: P63096), Gαs (UniProt ID: P63092), Hx (UniProt ID: P02790), and APC (UniProt ID: P04070) are available in the database, they were used directly for quality control without the requirement for any model generation.

### 4.2. Quality Assessments of the Structures

The quality of both the HMs and the AF-derived structures was examined by considering several parameters, including the stereochemical quality based on the overall and per-residue geometries, quality of non-bonded interactions, steric overlaps between non-bonded atoms, coherence between the 3D structure of the model and its amino acid sequence, and atomic and residue volumes in the protein structures. Various online validation servers, i.e., MolProbity [49], UCLA-DOE LAB, and SWISS-MODEL [50], were utilized for carrying out independent evaluations of the structures (Figure 1). The missing hydrogen atoms in the protein structures were added in the YASARA software (versions 18.2.7–21.8.27) before being used as input in the aforementioned validation tools, after which the structures were evaluated. MolProbity (version 4.5) is a structural analysis tool that provides information about the accuracy of macromolecules by evaluating their quality based on atomic contact analysis, geometry, and backbone torsion angles [51]. One of the main applications of MolProbity in this study was to determine the clashscores of residues in proteins calculated by the program Probe [52].

Regardless of the method by which the protein structure was generated, deviations from the correctly folded conformation may occur. The VERIFY3D tool [53], which checks the compatibility of 3D folded structures of proteins with their relative amino acid sequences, was used to identify local potential folding errors in addition to the general structures and to compare the accuracy of the folding of the generated HMs and AF structures. The geometry and stereochemical quality of the residues in the proteins were evaluated by using PROCHECK [54], since anomalies in certain stereochemical parameters, such as bond distances, torsion angles, and hydrogen bond energies, can also affect the atomic volumes in the residues, which is another parameter that contributes to the quality of a 3D structure [42]. The atomic-volume-associated evaluation of the structures, such as the conformation of the volume of an atom in the residue of the generated models to the overall standard volume of the same atom type was performed using the tool PROVE [42]. The residue-based determination of the correctly and incorrectly predicted regions based on their atomic interactions in comparison to standard values derived from highly resolved experimental structures was carried out by ERRAT [55]. A further model quality evaluation was performed using QMEANDisCo, a tool developed by SwissProt for assessing the absolute quality at both the local and the global level based on various geometrical features [56]. A general summary of the quality of the protein structures was obtained by using WHAT_CHECK [24]. Last, but not least, the confidence of the AF-predicted structures was additionally assessed by considering a metric called the predicted local distance difference test (pLDDT), which provides insights into how well the predicted models reconstructed the local atomic interactions in comparison to the pretrained experimental structures [8,11].

### 4.3. MD Simulation of Predicted Structures

In order to evaluate the behavior of the structures predicted by both methods in a physiological environment, the proteins were subjected to MD simulations. In this context, a group of parameters were assigned. The pH and the concentration of Na^+^ and Cl^-^ ions in the solution were adjusted to 7.4 and 0.9%, respectively. The simulations were conducted at 298 K (24.85 °C) with 0.997 g/mL water density under atmospheric pressure (NPT) fixed at 1 bar. The simulation cell was set in cubic with a width of 10 Å from all sides of the proteins, and the cell boundary was chosen as periodic. 

The predicted models, first, were exposed to a 500 ps refinement simulation by using the YAMBER force field [57] in YASARA in order to bring the predicted protein structures to their innate states. The resulting lowest-energy and high-quality structures obtained through conformational sampling of each protein were then used as input for 50 ns explicit MD simulations with the AMBER ff14SB [58] force field. The simulation trajectory of each protein was analyzed considering simulation parameters such as RMSD and RadGyration during the production phase. Furthermore, the structure in the most recent snapshot of the studied protein in the MD simulation trajectory was used as a representative structure for post-simulation evaluations (Figures S18 and S19).

## Figures and Tables

**Table 1:** Structural alignments of the homology models and AF structures with the experimentally determined structures.

Protein	Experimental Structure	Homology Model		AF Structure	
		RMSD (Å)	Seq. Identity (%)	RMSD (Å)	Seq. Identity (%)
	**5JS8**	1.05	100	0.81	97.41
**Gαi1**	**3UMS**	1.32	99.33	0.77	99.67
	**1Y3A**	1.33	99.29	0.74	100
	**6EG8**	0.98	100	0.83	99.71
**Gαs**	**7E5E**	1.05	98.71	0.90	100
	**6AU6**	1.28	97.18	0.99	99.08
**Hx**	**1QJS**	0.94	84.34	1.402	19.32
	**1AUT**	0.89	100	0.63	100
	**2AER**	1.35	40.67	1.106	42.58
**APC**	**3F6U**	0.90	100	1.03	40
	**1W0Y**	1.15	40.64	1.02	42.11
	**3HPT**	1.28	38.15	1.03	40
**Rap2**	**2RAP**	0.78	100	0.80	99.40
	**3RAP**	0.69	100	0.82	99.39
	**1AO6**	2.02	96.15	1.18	100
**HSA**	**1N5U**	1.30	100	1.84	96.33
	**4G03**	2.07	95.70	1.27	98.95
**IL-36α**	**6HPI**	1.57	100	1.83	100

**Note:** The alpha-carbon root-mean-square deviations (Cα-RMSD) and sequence identities between the generated HMs and the experimental structures used as templates, as well as between AF-predicted structures and the experimental structures for each protein group are shown.

**Table 2:** Comparison of the structural validation scores of the computationally predicted and the experimentally determined Gαi1 structures.

Protein	Validation Metric (or Method)	Homology Model *	AF *	5JS8 *	3UMS *	1Y3A *
**Gαi1**	**MolProbity**					
	Clashscore, all atoms (percentile)	0 (100th)	1.77 (99th)	0.97 (99th)	4.07 (99th)	12.94 (91th)
	Poor rotamers (%)	0.67	0	2.86	0.69	3.80
	Favored rotamers (%)	97.67	99.67	91.43	93.10	92.02
	Ramachandran outliers	0	0	0.62	0	0.34
	Rama-Z score	−0.65 ± 0.44	0.06 ± 0.42	−3.94 ± 0.39	−1.43 ± 0.43	−2.10 ± 0.40
	**Ramachandran plot (%)**					
	Most favored	89.6	95.2	84.8	92.0	93.1
	Additional allowed	10.1	4.8	13.9	8.0	6.5
	Generously allowed	0.3	0	0.7	0	0.4
	Disallowed	0	0	0.7	0	0
	Overall G-factors	0.17	0.21	−0.10	0.21	0.39
	**Verify3D (%)**					
	3D/1D profile	91.69	85.31	86.69	93.77	95.97
	**Errat (%)**					
	Overall quality factor	99.41	98.26	95.21	98.15	95.52
	**Prove (μ)**					
	Z-score	0.89 ± 26.80	1.16 ± 28.32	0.43 ± 1.32	0.23 ± 1.28	1.02 ± 29.01
	Z-score RMS	26.80	28.33	1.39	1.30	29.01
	**SwissProt**					
	QMEANDisCo global	0.76 ± 0.05	0.80 ± 0.05	0.74 ± 0.05	0.79 ± 0.05	0.82 ± 0.05

* The experimental structures (5JS8 [26], 3UMS [27], 1Y3A [28]), the HM generated from the experimental structures, and the structures predicted by AF were specifically evaluated.

**Table 3:** Comparison of the structural validation scores of the computationally predicted and the experimentally determined Gαs structures.

Protein	Validation Method	Homology Model *	AF *	6EG8 *	7E5E *	6AU6 *
**Gαs**	**MolProbity**					
	Clashscore, all atoms (percentile)	0 (100th)	2.20 (99th)	5.71 (97th)	0.18 (100th)	1.25 (99th)
	Poor rotamers (%)	1.47	0	0	0.66	0.65
	Favored rotamers	98.42	98.86	89.29	97.04	97.73
	Ramachandran outliers	0	0	0	0	0.29
	Rama-Z score	−0.24 ± 0.38	0.15 ± 0.41	−2.85 ± 0.36	−0.24 ± 0.43	−0.25 ± 0.44
	**Ramachandran plot (%)**					
	Most favored	93.4	93.6	91.6	94.2	90.2
	Additional allowed	6.6	6.4	8.4	5.8	9.2
	Generously allowed	0	0	0	0	0.6
	Disallowed regions	0	0	0	0	0
	Overall G-factors	0.27	0.21	0.21	0.21	0.22
	**Verify3D (%)**					
	3D/1D profile	87.66	87.82	83.60	94.38	99.42
	**Errat (%)**					
	Overall quality factor	99.73	98.67	96.10	99.70	99.09
	**Prove (μ)**					
	Z-score	0.37 ± 1.81	0.35 ± 1.17	0.39 ± 1.20	0.36 ± 1.20	0.09 ± 1.21
	Z-score RMS	1.24	1.21	1.26	1.25	1.22
	**SwissProt**					
	QMEANDisCo global	0.77 ± 0.05	0.77 ± 0.05	0.77 ± 0.05	0.77 ± 0.05	0.79 ± 0.05

* The experimental structures (6EG8 [29], 7E5E [30], 6AU6 [31]), the HM generated from the experimental structures, and the structures predicted by AF were specifically evaluated.

**Table 4:** Comparison of the structural validation scores of the computationally predicted and the experimentally determined APC structures.

Protein	Validation Data	Homology Model *	AF *	1AUT *	2AER *	3F6U *	1W0Y *	3HPT *
**APC**	**MolProbity**							
	Clashscore, all atoms (percentile)	0 (100th)	1.39 (99th)	22.49 (86th)	17.06 (40th)	15.26 (95th)	44.14 16th)	33.37 (15th)
	Poor rotamers (%)	2.28	1.24	11.42	3.15	14.48	2.77	2.08
	Favored rotamers (%)	94.59	95.02	77.85	93.31	73.79	91.70	0.01
	Ramachandran outliers (%)	0.50	2.61	0.30	0.54	2.10	0.55	0
	Rama. distr. Z-score	−1.08 ± 0.38	−1.59 ± 0.37	−2.98 ± 0.38	−0.58 ± 0.34	−3.10 ± 0.40	−2.05 ± 0.31	−0.62 ± −0.30
	**Ramachandran plot (%)**							
	Most favored regions	89.5	80.7	84.3	88.8	80.1	86.8	85.8
	Additional allowed regions	9.7	17.5	15.7	10.6	19.9	12.6	13.9
	Generous. allowed regions	0.6	1.5	0.0	0.4	0.0	0.4	0.4
	Disallowed regions	0.3	0.2	0.0	0.2	0.0	0.2	0.0
	Overall G-factors	0.08	−0.04	0.14	0.19	−0.46		
	**Verify3D (%)**							
	3D/1D profile	85.68	72.02	95.05	93.69	95.40	92.93	91.76
	**Errat (%)**							
	Overall quality factor	94.33	95.81	85.47	92.18	84.93	9.98	95.96
	**Prove (μ)**							
	Z-score	1.12 ± 26.87	0.44 ± 1.30	0.96 ± 26.43	1.22 ± 35.12	0.98 ± 26.52	1.80 ± 42.15	2.742 ± 52.014
	Z-score RMS	26.88	1.38	26.44	35.14	26.51	42.18	52.078
	**SwissProt**							
	QMEANDisCo global	0.74 ± 0.05	0.67 ± 0.05	0.87 ± 0.05	0.86 ± 0.05	0.86 ± 0.05	0.85 ± 0.05	0.85 ± 0.05

* The experimental structures (1AUT [32], 2AER [33], 3F6U [34], 1W0Y [35], and 3HPT [36]), the HM generated from the experimental structures, and the structures predicted by AF were specifically evaluated.

**Table 5:** Comparison of the structural validation scores of the computationally predicted and the experimentally determined hemopexin structures.

Protein	Validation Data	Homology Model *	AF *	1QJS *
**Hemopexin**	**MolProbity**			
	Clashscore, all atoms	0 (100th)	2.11 (99th)	15.46 (96th)
	Poor rotamers (%)	0.84	1.56	10.80
	Favored rotamers (%)	97.21	95.05	79.55
	Ramachandran outliers (%)	0.24	4.13	0.99
	Rama. distribution Z-score	−0.69 ± 0.38	−1.74 ± 0.35	−3.0 ± 0.25
	**Ramachandran plot (%)**			
	Most favored regions	90.3	83.6	82.7
	Additional allowed regions	8.9	12.4	15.8
	Generously allowed regions	0.3	2.1	1.2
	Disallowed regions	0.6	1.8	0.3
	Overall G-factors	0.07	−0.20	−0.20
	**Verify3D (%)**			
	3D/1D profile	95.77	90.26	99.75
	**Errat (%)**			
	Overall quality factor	79.42	82.86	72.31
	**Prove (μ)**			
	Z-score	1.09 ± 24.39	0.56 ± 1.32	0.49 ± 1.31
	Z-score RMS	24.41	1.44	1.40
	**SwissProt**			
	QMEANDisCo global	0.81 ± 0.05	0.78 ± 0.05	0.91 ± 0.05

* The experimental structure (1QJS [15]), the HM generated from the experimental structure, and the structure predicted by AF were specifically evaluated.

**Table 6:** Comparison of the structural validation scores of the computationally predicted and the experimentally determined Rap2 structures.

Protein	Validation Data	Homology Model *	AF *	2RAP *	3RAP *
**Rap2**	**MolProbity**				
	Clashscore, all atoms	0 (100th)	1.39 (100th)	4.44 (99th)	2.59 (100th)
	Poor rotamers (%)	0.64	0	5.41	3.38
	Favored rotamers (%)	98.09	97.53	85.14	91.22
	Ramachandran outliers (%)	0	0.55	0.61	1.21
	Rama. distribution Z-score	−0.59 ± 0.59	−0.29 ± 0.62	−2.41 ± 0.56	−1.16 ± 0.55
	**Ramachandran plot (%)**				
	Most favored regions	93.0	90.2	89.3	90.6
	Additional allowed regions	6.4	9.2	10.7	7.4
	Generously allowed regions	0.0	0.6	0.0	2.0
	Disallowed regions	0.6	0.0	0.0	0.0
	Overall G-factors	0.20	0.08	−0.18	−0.04
	**Verify3D (%)**				
	3D/1D profile	58.19	47.54	53.29	61.68
	**Errat (%)**				
	Overall quality factor	95.65	98.16	93.96	98.68
	**Prove (μ)**				
	Z-score	-	-	-	-
	Z-score RMS	-	-	-	-
	**SwissProt**				
	QMEANDisCo global	0.83 ± 0.07	0.83 ± 0.06	0.88 ± 0.07	0.87 ± 0.07

* The experimental structures (2RAP [37] and 3RAP [17]), the HM generated from the experimental structures, and the structures predicted by AF were specifically evaluated.

**Table 7:** Comparison of the structural validation scores of the computationally predicted and the experimentally determined HSA structures.

Protein	Validation Data	Homology Model *	AF *	1AO6 *	1N5U	4G03
**HSA**	**MolProbity**					
	Clashscore, all atoms	0.21 (100th)	2.07 (99th)	13.92 (86th)	21.97 (23rd)	6.91 (97th)
	Poor rotamers (%)	6 (1.16)	3 (0.56)	24 (4.74)	18 (3.54)	27 (5.34)
	Favored rotamers (%)	501 (96.72)	522 (97.94)	436 (86.17)	465 (91.36)	431 (85.18)
	Ramachandran outliers (%)	2 (0.34)	0 (0.00)	11 (1.91)	6 (1.03)	5 (0.87)
	Rama. distribution Z-score	0.74 ± 0.33	0.41 ± 0.32	−4.28 ± 0.28	−0.43 ± 0.32	−2.69 ± 0.30
	**Ramachandran plot (%)**					
	Most favored regions	93.9	94.9	88.5	93.2	90.4
	Additional allowed regions	5.2	5.1	11.5	5.7	9.1
	Generously allowed regions	0.7	0.0	0.0	0.9	0.2
	Disallowed regions	0.2	0.0	0.0	0.2	0.4
	Overall G-factors	0.33	0.24	0.21	0.44	0.18
	**Verify3D (%)**					
	3D/1D profile	79.12	72.41	74.18	79.55	79.38
	**Errat (%)**					
	Overall quality factor	98.29	97.63	93.26	98.08	96.47
	**Prove (μ)**					
	Z-score	-	-	-	-	-
	Z-score RMS	-	-	-	-	-
	**SwissProt**					
	QMEANDisCo global	0.81 ± 0.05	0.84 ± 0.05	0.81 ± 0.05	0.82 ± 0.05	0.83 ± 0.05

* The experimental structures (1AO6 [18], 15NU [38] and 4G03 (https://www.rcsb.org/structure/4G03, accessed on 6 November 2023), the HM generated from the experimental structures, and the structures predicted by AF were specifically evaluated.

**Table 8:** Comparison of the structural validation scores of the computationally predicted and the experimentally determined IL-36α structures.

Protein	Validation Data	Homology Model *	AF *	6HPI *
**IL-36α**	**MolProbity**			
	Clashscore, all atoms	0 (100th)	1.61 (99th)	7.23 (86th)
	Poor rotamers (%)	3 (2.14)	0 (0.00)	29 (20.71)
	Favored rotamers (%)	135 (96.43)	139 (99.29)	80 (57.14)
	Ramachandran outliers (%)	1 (0.64)	0 (0.00)	3 (1.92)
	Rama. distribution Z-score	0.38 ± 0.67	−0.79 ± 0.59	−4.62 ± 0.55
	**Ramachandran plot (%)**			
	Most favored regions	89.7	89.7	73.5
	Additional allowed regions	10.3	10.3	25.7
	Generously allowed regions	0	0	0.7
	Disallowed regions	0	0	0
	Overall G-factors	0.03	0.03	−0.16
	**Verify3D (%)**			
	3D/1D profile	70.25	70.25	59.49
	**Errat (%)**			
	Overall quality factor	90.90	90.90	85.18
	**Prove (μ)**			
	Z-score	-	-	-
	Z-score RMS	-	-	-
	**SwissProt**			
	QMEANDisCo global	0.76 ± 0.07	0.71 ± 0.07	0.90 ± 0.07

* The experimental structure (6HPI [39]), the HM generated from the experimental structures, and the structures predicted by AF were specifically evaluated.

**Table 9:** Experimental data of the template structures used as the input for the homology models of the proteins.

PDB	Protein	Ligand(s)	Resolution	Released Date (Updated)	Sequence Length	Organism	Mutation(s)
**5JS8**	Gαi1	GDP	NMR ensemble	2016 (2019)	326	*Homo sapiens*	−
**3UMS**	Gαi1	GDP, SO_4_^2−^, Cl^−^	2.34 Å	2012 (2012)	354	*Homo sapiens*	+
**1Y3A**	Gαi1	GDP	2.50 Å	2005 (2019)	329	*Homo sapiens*	−
**6EG8**	Gαs	GDP, Mg^2+^	2.80 Å	2019 (2019)	381	*Homo sapiens*	−
**7E5E**	Gαs	GDP, Cl^−^	1.95 Å	2022 (2022)	348	*Homo sapiens*	−
**6AU6**	Gαs	GDP, Cl^−^, Mg^2+^, GOL	1.70 Å	2018 (2019)	377	*Homo sapiens*	+
**1QJS**	Hemopexin	HEM, PO_4_^3−^, Cl^−^, Na^+^	2.90 Å	2000 (2019)	460	*Oryctolagus* *cuniculus*	−
**1AUT**	APC	0G6, BHD	2.80 Å	1996 (2013)	364	*Homo sapiens*	−
**3F6U**	APC	0G6, Ca^2+^, Na^+^	2.80 Å	2008 (2013)	338	*Homo sapiens*	−
**2AER**	Factor VIIa	GLC, FUC, BEN, Zn^2+^, Ca^2+^, Cl^−^, Na^+^, Mg^2+^,	1.87 Å	2005 (2020)	396	*Homo sapiens*	+
**1W0Y**	Factor VIIa	771, BGC, FUC, CAC, Ca^2+^	2.50 Å	2004 (2020)	396	*Homo sapiens*	−
**3HPT**	Factor X	YET, MES, GOL, DMS, ACT, Ca^2+^, Na^+^	2.19 Å	2009 (2017)	332	*Homo sapiens*	−
**2RAP**	Rap2	GTP, Mg^2+^	2.60 Å	1998 (2011)	167	*Homo sapiens*	−
**3RAP**		GTP, Mg^2+^	2.20 Å	1999 (2023)	167	*Homo sapiens*	−
**6HPI**	IL-36α	-	NMR ensemble	2019 (2023)	158	*Homo sapiens*	−
**1AO6**	HSA	-	2.50 Å	1998 (2011)	585	*Homo sapiens*	−
**1N5U**	HSA	HEM, MYR	1.90 Å	2003 (2011)	585	*Homo sapiens*	−
**4G03**	HSA	-	2.22 Å	2013 (2013)	585	*Homo sapiens*	−

**Note:** 5JS8 (10 conformers submitted) [26], 3UMS [27], and 1Y3A [28] are experimentally determined structures of Gαi1 that were used as templates for the generation of Gαi1 HM [13]. In the construction of the Gαs HM, the chain I of 6EG8 [29], 7E5E [30], and 6AU6 [31] were used as inputs. Chain A of 1QJS [15] was the only exploited template structure for the construction of the Hx HM [14]. Five crystal structures, 1AUT [32], 2AER [33], 3F6U [34], 1W0Y [35], and 3HPT [36], were fed in as inputs to construct the APC HM [16]. 2RAP [37] and 3RAP [17] were the crystal structures utilized in the generation of Rap2 HM. In the generation of IL-36α HM, 6HPI (20 conformers submitted) [39] was the only experimental structure. Three crystal structures, 1AO6 [18], 1N5U [38], and 4G03 (https://www.rcsb.org/structure/4G03, accessed on 6 November 2023), were used in the prediction of HSA HM.

## Data Availability

Data is contained within the article and Supplementary Materials.

## Supplementary Materials

The following supporting information can be downloaded at: https://www.mdpi.com/article/10.3390/ph16121662/s1, Figure S1: Structural alignments of the homology models and AF structures with the experimentally determined structures; Figure S2: Structural alignments of the homology models and AF structures with the experimentally determined structures; Figure S3: Verify3D plots for the Gαi1 structures; Figure S4: ERRAT plots for the Gαi1 structures; Figure S5: Verify3D plots for the Gαs structures; Figure S6: ERRAT plots for the Gαs structures; Figure S7: Verify3D plots for the APC structures; Figure S8: ERRAT plots for the APC structures; Figure S9: Verify3D plots for the hemopexin structures; Figure S10: ERRAT plots for the hemopexin structures; Figure S11: Verify3D plots for the Rap2 structures; Figure S12: ERRAT plots for the Rap2 structures; Figure S13: Verify3D plots for the HSA structures; Figure S14: ERRAT plots for the HSA structures; Figure S15: Verify3D plots for the IL-36α structures; Figure S16: ERRAT plots for the IL-36α structures; Figure S17: Structural alignments of the pre- and post-MD simulated structures from AF; Figure S18: Selection of the representative post MD simulation structure for nucleotide binding proteins; Figure S19: Selection of the representative post MD simulation structure for heme binding proteins; Table S1: AlphaFold pLDDT scores of functionally relevant regions of select proteins; Table S2: Comparison of the structural validation scores of the MD simulated AF-predicted model of Gαi1 with the HM and pure AF-predicted model; Table S3: Comparison of the structural validation scores of the MD simulated AF-predicted model of Gαs with the HM and pure AF-predicted model; Table S4: Comparison of the structural validation scores of the MD simulated AF-predicted model of APC with the HM and pure AF-predicted model; Table S5: Comparison of the structural validation scores of the refined AF-predicted model of hemopexin with the HM and unrefined AF-predicted model. Table S6: Comparison of the structural validation scores of the refined AF-predicted model of HSA with the HM and unrefined AF-predicted model; Table S7: Comparison of the structural validation scores of the refined AF-predicted model of Rap2 with the HM and unrefined AF-predicted model; Table S8: Comparison of the structural validation scores of the refined AF-predicted model of IL-36α with the HM and unrefined AF-predicted model.

## Abbreviations

AF, AlphaFold; AI, artificial intelligence; APC, activated protein C; Gαi1, G protein αi1 subunit; Gαs, G protein αs subunit; HAS, human serum albumin; HM, homology model; IL-36α, interleukin-36α; MD, molecular dynamics; MSA, multiple sequence alignment; NMR, nuclear magnetic resonance; RadGyration, radius of gyration; Rap2, Ras-related protein; RMSD, root-mean-square deviation; RMSDBb, backbone root-mean-square deviation; SW, switch; Z-score rms, Z-score root-mean-square deviation.
