# Supplementary material for: Quality Assessment of Selected Protein Structures Derived from Homology Modeling and AlphaFold
Source: Pharmaceuticals (Basel). 2023 Nov 29;16(12):1662. doi: 10.3390/ph16121662 (PMC10747200; doi:10.3390/ph16121662)
Supplement: Supplementary file 1 [file pharmaceuticals-16-01662-s001.zip › pharmaceuticals-2733232-supplementary.pdf]

Table S1. AlphaFold pLDDT score of functionally relevant regions of select proteins.

Protein	Region/Motifs	pLDDT Score
Gαi1	SWI	90.73
	SWII	94.13
	SWIII	96.26
Gαs	SWI	95.24
	SWII	94.69
	SWIII	95.39
Hemopexin	VWKSHKWDR	87.66
	FRQGHNSVF	92.55
	PGRGHGHRN	69
	RGHGHRNGT	56
	RCSPHLVLS	91
	RDGWHSWPI	94
APC	WIHGHIRDK	94.66
	TGWGYHSSR	64.88
Rap2	SWI	89.75
	SWII	90.96
HSA	IARRHPYFYAPEL	98.18
	FAKRYKAAF	97.69
IL 36α	FLFYHSQSG	97.22
	SEGGCPLIL	89.66

Model Confidence: Very high (pLDDT > 90), Confident (90 > pLDDT > 70), Low (70 > pLDDT > 50), Very low (pLDDT < 50)

Table S2. Comparison of the structural validation scores of the MD simulated AF-predicted model of Gαi1 with the HM and pure AF-predicted model.

Protein	Validation Method	HM Pre-Simulation	HM Post-Simulation	AF Pre-Simulation	AF Post-Simulation
Gαi1	**MolProbity**				
	Clashscore, all atoms (percentile)	0 (100th)	0 (100th)	1.77 (99th)	1.24 (99th)
	Poor rotamers (%)	0.67	0.67	0	1.64
	Favored rotamers (%)	97.67	98.56	99.67	95.74
	Ramachandran outliers	0	0	0	0.57
	Rama-Z score	−0.65 ± 0.44	−0.65 ± 0.44	0.06 ± 0.42	−3.43 ± 0.41
	**Ramachandran plot (%)**				
	Most favored	89.6	89.6	95.2	90.3
	Additional allowed	10.1	10.1	4.8	9.7
	Generously allowed	0.3	0.3	0	0.0
	Disallowed	0	0	0	0
	Overall G-factors	0.17	0.17	0.21	−0.71
	**Verify3D (%)**				
	3D/1D profile	91.69	77.65	85.31	72.32
	**Errat (%)**				
	Overall quality factor	99.41	99.41	98.26	93.67
	**Prove (μ)**				
	Z-score	0.89 ± 26.80	-	1.16 ± 28.32	-
	Z-score RMS	26.80	-	28.33	-
	**SwissProt**				
	QMEANDisCo global	0.76 ± 0.05	0.75 ± 0.05	0.80 ± 0.05	0.73 ± 0.05

Table S3. Comparison of the structural validation scores of the MD simulated AF-predicted model of Gαs with the HM and pure AF-predicted model.

Protein	Validation Method	HM Pre-Simulation	HM Post-Simulation	AF Pre-Simulation	AF Post-Simulation
	**MolProbity**				
	Clashscore, all atoms (percentile)	0 (100th)	0.16 (100th)	2.20 (99th)	1.26 (99th)
	Poor rotamers (%)	1.47	0.59	0	1.43
	Favored rotamers (%)	98.42	98.82	98.86	93.88
	Ramachandran outliers	0	0.0	0	1.02
	Rama-Z score	−0.24 ± 0.38	−0.63 ± 0.39	0.15 ± 0.41	−3.49 ± 0.36
	**Ramachandran plot (%)**				
	Most favored	93.4	92.9	93.6	90.6
	Additional allowed	6.6	7.1	6.4	8.9
	Generously allowed	0	0	0	0.6
**Gαs**	Disallowed	0	0	0	0
	Overall G-factors	0.27	0.25	0.21	−0.70
	**Verify3D (%)**				
	3D/1D profile	87.66	80.05	87.82	74.37
	**Errat (%)**				
	Overall quality factor	99.73	100	98.67	90.71
	**Prove (μ)**				
	Z-score	0.37 ± 1.81	-	0.35 ± 1.17	-
	Z-score RMS	1.24	-	1.21	-
	**SwissProt**				
	QMEANDisCo global	0.77 ± 0.05	0.75 ± 0.05	0.77 ± 0.05	0.69 ± 0.05

Table S4. Comparison of the structural validation scores of the MD simulated AF-predicted model of APC with the HM and pure AF-predicted model.

Protein	Validation Method	HM Pre-Simulation	HM Post-Simulation	AF Pre-Simulation	AF Post-Simulation
APC	**MolProbity**				
	Clashscore, all atoms(percentile)	0 (100th)	1.28	1.39 (99th)	1.67(99th)
	Poor rotamers (%)	2.28	0(0.00)	1.24	3(0.75)
	Favored rotamers	94.59	-	95.02	370(92.04)
	Ramachandran outliers	0.50	4(1.00)	2.61	6(1.31)
	Rama-Z score	−1.08 ± 0.38	−3.89 ± 0.33	−1.59 ± 0.37	−3.28 ± 0.31
	**Ramachandran plot (%)**				
	Most favored	89.5	82.1	80.7	83.2
	Additional allowed	9.7	17.0	17.5	15.6
	Generously allowed	0.6	0.6	1.5	1.0
	Disallowed regions	0.3	0.3	0.2	0.2
	Overall G-factors	0.08	−0.86	−0.04	−0.83
	**Verify3D (%)**				
	3D/1D profile	85.68	62.72	72.02	65.94
	**Errat (%)**				
	Overall quality factor	94.33	85.38	95.81	83.80
	**Prove (μ)**				
	Z-score	-	-	-	-
	Z-score RMS	-	-	-	-
	**SwissProt**				
	QMEANDisCo global	0.74 ± 0.05	0.64 ± 0.05	0.67 ± 0.05	0.64 ± 0.05

Table S5. Comparison of the structural validation scores of the refined AF-predicted model of Hemopexin with the HM and unrefined AF-predicted model.

Protein	Validation Method	HM Pre-Simulation	HM Post-Simulation	AF Pre-Simulation	AF Post-Simulation
Hx	**MolProbity**				
	Clashscore, all atoms(percentile)	0(100th)	1.97(100th)	2.11(99th)	1.83(99th)
	Poor rotamers (%)	0.84	10(2.79)	1.56	10(2.60)
	Favored rotamers	97.21	316(88.27)	95.05	340(88.54)
	Ramachandran outliers	0.24	7(1.65)	4.13	4(0.87)
	Rama-Z score	−0.69 ± 0.38	−3.52 ± 0.34	−1.74 ± 0.35	−2.30 ± 0.33
	**Ramachandran plot (%)**				
	Most favored	90.3	81.1	83.6	88.1
	Additional allowed	8.9	18.3	12.4	10.6
	Generously allowed	0.3	0.3	2.1	0.8
	Disallowed regions	0.6	0.3	1.8	0.5
	Overall G-factors	0.07	−0.95	−0.20	−0.88
	**Verify3D (%)**				
	3D/1D profile	95.77	96.95	90.26	90.26
	**Errat (%)**				
	Overall quality factor	79.42	68.93	82.86	76.44
	**Prove (μ)**				
	Z-score	-	-	-	-
	Z-score RMS	-	-	-	-
	**SwissProt**				
	QMEANDisCo global	0.81 ± 0.05	0.72 ± 0.05	0.78 ± 0.05	0.72 ± 0.0.05

Table S6. Comparison of the structural validation scores of the refined AF-predicted model of HSA with the HM and unrefined AF-predicted model.

Protein	Validation Method	HM Pre-Simulation	HM Post-Simulation	AF Pre-Simulation	AF Post-Simulation
HSA	**MolProbity**				
	Clashscore, all atoms(percentile)	0.21(100th)	1.6(100th)	2.07(99th)	1.55(99th)
	Poor rotamers (%)	6(1.16)	8(1.54)	3(0.56)	15(2.81)
	Favored rotamers	501(96.72)	476(91.89)	522(97.94)	487(91.37)
	Ramachandran outliers	2(0.34)	2(0.34)	0(0.00)	4(0.66)
	Rama-Z score	0.74 ± 0.33	−3.92 ± 0.28	0.41 ± 0.32	−4.22 ± 0.26
	**Ramachandran plot (%)**				
	Most favored	93.9	92.1	94.9	90.9
	Additional allowed	5.2	7.6	5.1	9.1
	Generously allowed	0.7	0.4	0.0	0
	Disallowed regions	0.2	0	0.0	0
	Overall G-factors	0.33	−0.62	0.24	−0.65
	**Verify3D (%)**				
	3D/1D profile	79.12	75.42	72.41	76.35
	**Errat (%)**				
	Overall quality factor	98.29	94.67	97.63	93.83
	**Prove (μ)**				
	Z-score	-	-	-	-
	Z-score RMS	-	-	-	-
	**SwissProt**				
	QMEANDisCo global	0.81 ± 0.05	0.72 ± 0.05	0.84 ± 0.05	0.76 ± 0.05

Table S7. Comparison of the structural validation scores of the refined AF-predicted model of Rap2 with the HM and unrefined AF-predicted model.

Protein	Validation Method	HM Pre-Simulation	HM Post-Simulation	AF Pre-Simulation	AF Post-Simulation
Rap2	**MolProbity**				
	Clashscore, all atoms (percentile)	0 (100th)	0.36 (100th)	1.39(100th)	1.04 (99th)
	Poor rotamers (%)	0.64	1.27	0	2.47
	Favored rotamers (%)	98.09	93.63	97.53	92.82
	Ramachandran outliers	0	0.57	0.55	0.55
	Rama-Z score	−0.59 ± 0.59	−3.90 ± 0.51	−0.29 ± 0.62	−3.15 ± 0.51
	**Ramachandran plot (%)**				
	Most favored	93.0	86.0	90.2	90.8
	Additional allowed	6.4	12.7	9.2	6.7
	Generously allowed	0.0	1.3	0.6	1.8
	Disallowed	0.6	0	0.0	0.6
	Overall G-factors	0.20	−0.74	0.08	−0.73
	**Verify3D (%)**				
	3D/1D profile	58.19	68.36	47.54	61.20
	**Errat (%)**				
	Overall quality factor	95.65	94.26	98.16	82.08
	**Prove (μ)**				
	Z-score	-	-	-	-
	Z-score RMS	-	-	-	-
	**SwissProt**				
	QMEANDisCo global	0.83 ± 0.07	0.76 ± 0.07	0.83 ± 0.06	0.75 ± 0.06

Table S8. Comparison of the structural validation scores of the refined AF-predicted model of IL 36α with the HM and unrefined AF-predicted model.

Protein	Validation Method	HM Pre-Simulation	HM Post-Simulation	AF Pre-Simulation	AF Post-Simulation
IL-36α	**MolProbity**				
	Clashscore, all atoms(percentile)	0(100th)	1.2(99th)	1.61(99th)	0.8(99th)
	Poor rotamers (%)	3(2.14)	2(1.43)	0(0.00)	8(5.71)
	Favored rotamers	135(96.43)	129(92.14)	139(99.29)	119(85.00)
	Ramachandran outliers	1(0.64)	1(0.64)	0(0.00)	2(1.28)
	Rama-Z score	0.38 ± 0.67	−2.78 ± 0.61	−0.79 ± 0.59	−3.35 ± 0.52
	**Ramachandran plot (%)**				
	Most favored	89.7	85.3	89.7	85.3
	Additional allowed	10.3	13.2	10.3	13.2
	Generously allowed	0	0.7	0	0.7
	Disallowed regions	0	0.7	0	0.7
	Overall G-factors	0.03	−0.92	0.03	−0.92
	**Verify3D (%)**				
	3D/1D profile	70.25	62.03	70.25	62.03
	**Errat (%)**				
	Overall quality factor	90.90	83.82	90.90	83.82
	**Prove (μ)**				
	Z-score	-	-	-	-
	Z-score RMS	-	-	-	-
	**SwissProt**				
	QMEANDisCo global	0.76 ± 0.07	0.62 ± 0.07	0.71 ± 0.07	0.67 ± 0.07

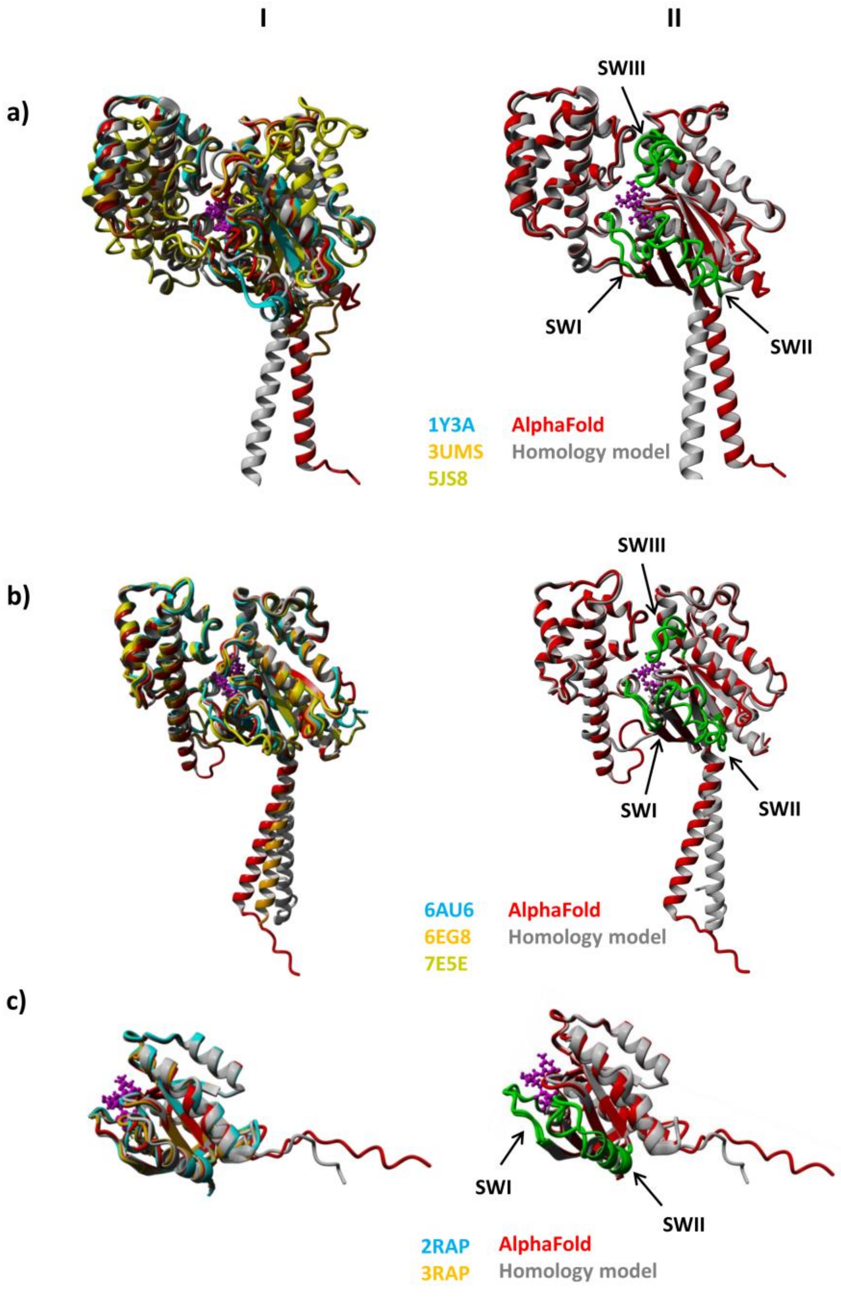

Figure S1. Structural alignments of the homology models and AF structures with the experimentally determined structures. The structural alignments of Gαi1, Gαs, and Rap2 are shown in panels **a**, **b**, and **c**, respectively. Column **I** show the alignment of the computationally generated and experimentally determined models (structures used and their PDB IDs are indicated by specific colors), whereas column **II** demonstrates the alignment of the HM (gray) and the AF structure (red). In each panel, the nucleotide, which could not be predicted by AF, in the binding pocket is shown in magenta color in ball & stick style. Secondary structural elements, particularly the α-helices other than the N-terminal helix, in Gαi1 structures (panel **a**, **I**) show irregular alignment, while the alignment of these regions in Gαs structures is more ordered (panel **b**, **I**). The RMSD between the Gαi1 HM and the AF structure (panel **a**, **II**) is 1.17Å, while the RMSD between the Gαs structures (panel **b**, **II**) is 0.99Å. Moreover, calculated RMSD between Rap2 HM and the AF structure (panel **c**, III) is 1.016 Å. The deviations are caused by the switch (SW) regions (highlighted as green) and other loop regions.

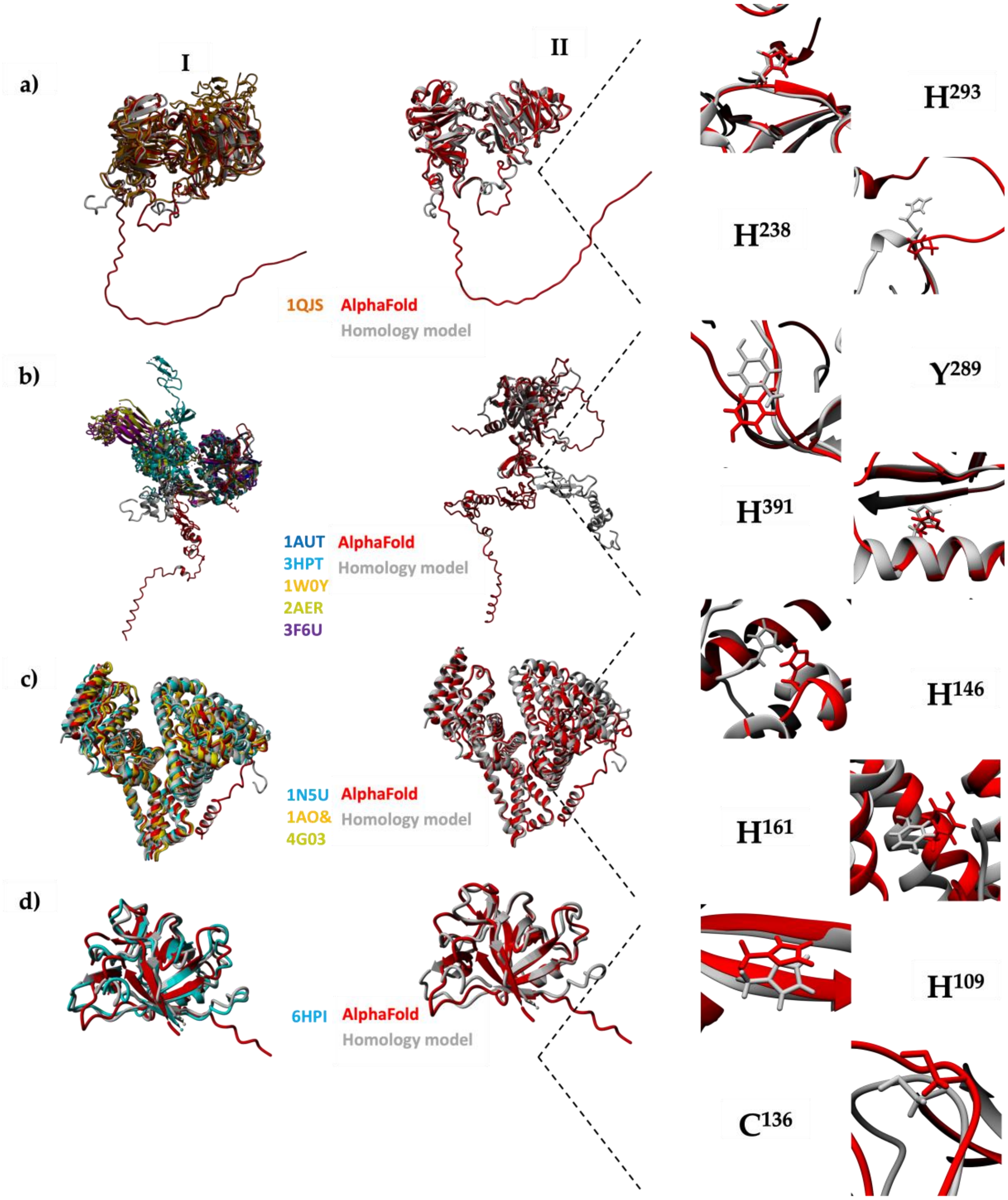

Figure S2. Structural alignments of the homology models and AF structures with the experimentally determined structures**.** The structural alignments of Hx, APC, HSA, and IL-36α were shown in panels **a**, **b**, **c**, and **d**, respectively. Column **I** shows the alignment of the computationally generated and experimentally determined models (structures used and their PDB IDs are indicated by specific colors), whereas column II demonstrates the alignment of the HM (gray) and the AF structure (red). Secondary structural elements in Hemopexin structures (panel **a**, **I**) show reasonable alignment, while the alignment of these regions in APC structures looks more erratic (panel **b, I**). The RMSD between the hemopexin HM and the AF structure (panel **a**, **II**) is 0.97Å, while the RMSD between the APC structures (panel **b**, **II**) is 0.92Å. The calculated RMSD between HSA HM and the AF structure (panel **c, III**) and IL-36α HM and the AF structure (panel **d**, **IV**) are 1.731Å and 1.604Å, respectively.

a)

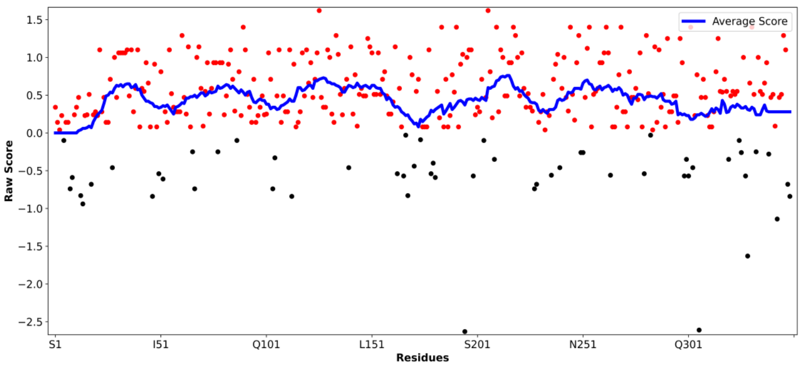

b)

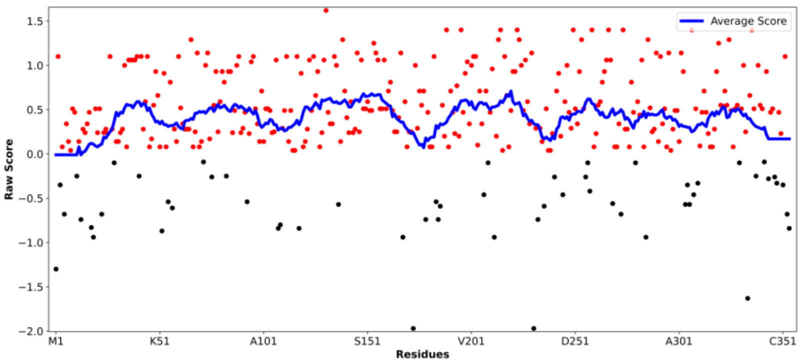

Figure S3. Verify3D plots for the Gαi1 structures**.** The average (blue line) and raw scores (red and black dots) of the residues for HM **(a)** and AF structure **(b) were** shown. Red dots indicate residues with a raw value above zero, while black dots point out the residues with negative values.

a)

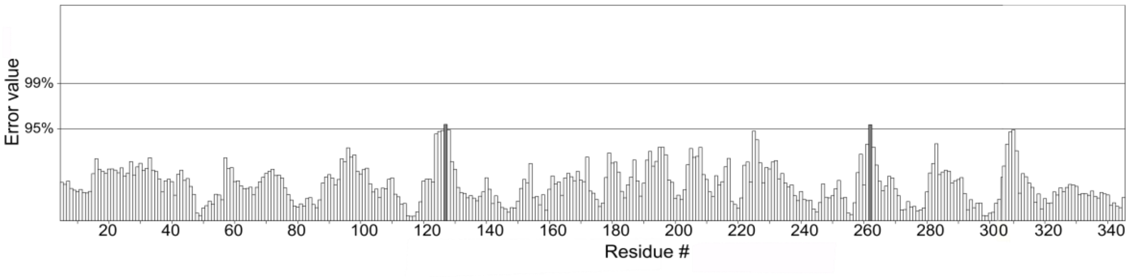

b)

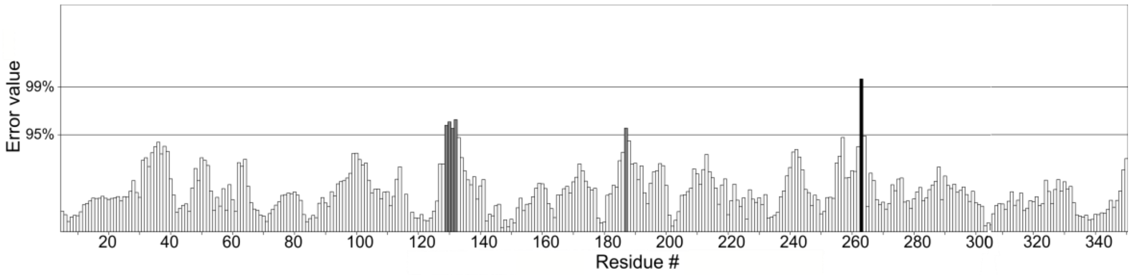

Figure S4. ERRAT plots for the Gαi1 structures**.** The error rates of the **(a)** HM and **(b)** AF structure were illustrated. White bars represent normal residues in the structure. Gray bars display residues with error rates between 90-95%, while black bars demonstrate misfolded regions with high error (>99%).

a)

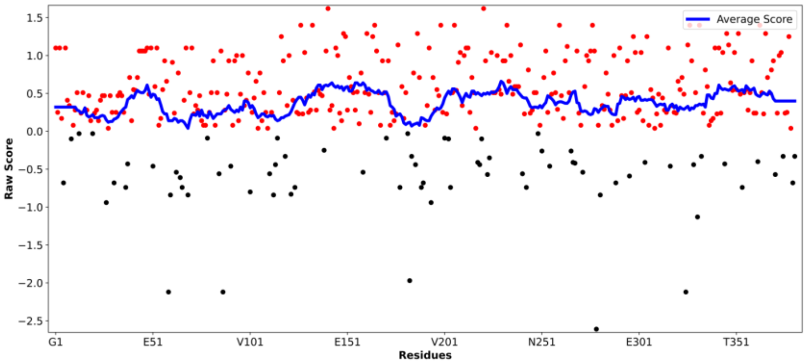

b)

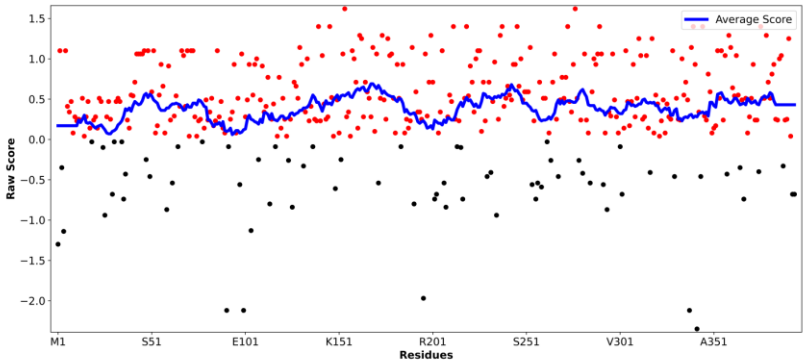

Figure S5. Verify3D plots for the Gαs structures. The average (blue line) and raw scores (red and black dots) of the residues for HM **(a)** and AF structure **(b)** were shown. Red dots indicate residues with a raw value above zero, while black dots point out the residues with negative values.

a)

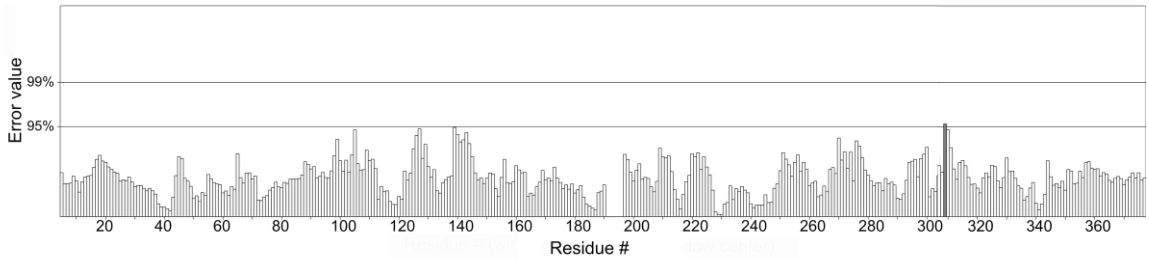

b)

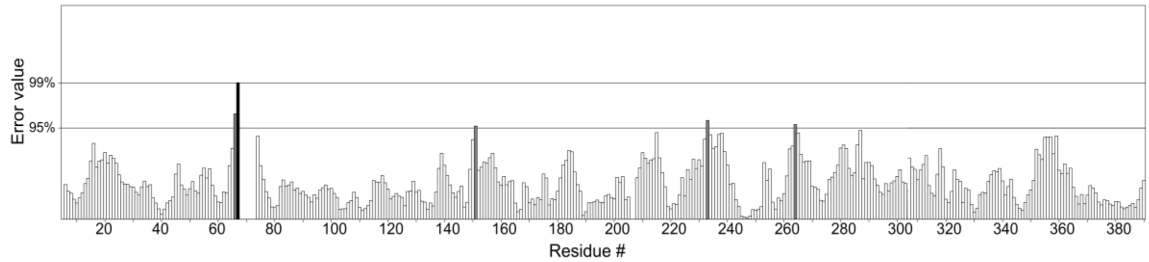

Figure S6. ERRAT plots for the Gαs structures. The error rates of HM **(a)** and AF structure **(b)** are illustrated. White bars represent normal residues in the structure. Gray bars display residues with error rates between 90-95%, while black bars demonstrate misfolded regions with high error (>99%).

a)

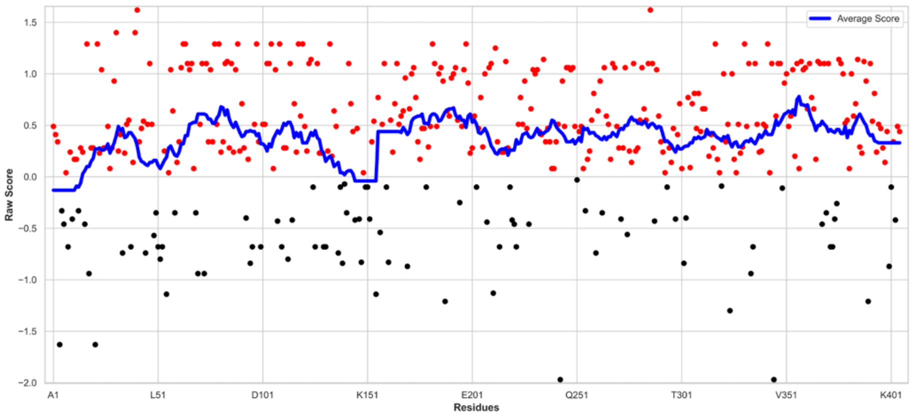

b)

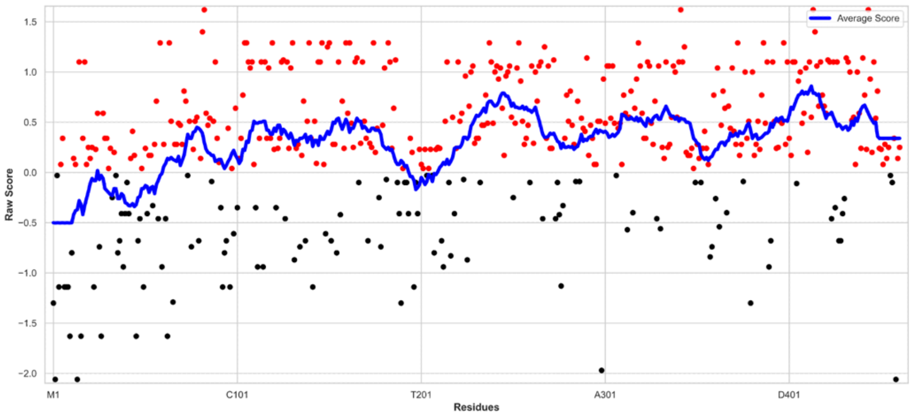

Figure S7. Verify3D plots for the APC structures**.** The average (blue line) and raw scores (red and black dots) of the residues for HM chain A **(a),** HM chain B **(b)** and AF structure **(c)** were shown. Red dots indicate residues with a raw value above zero, while black dots point out the residues with negative values.

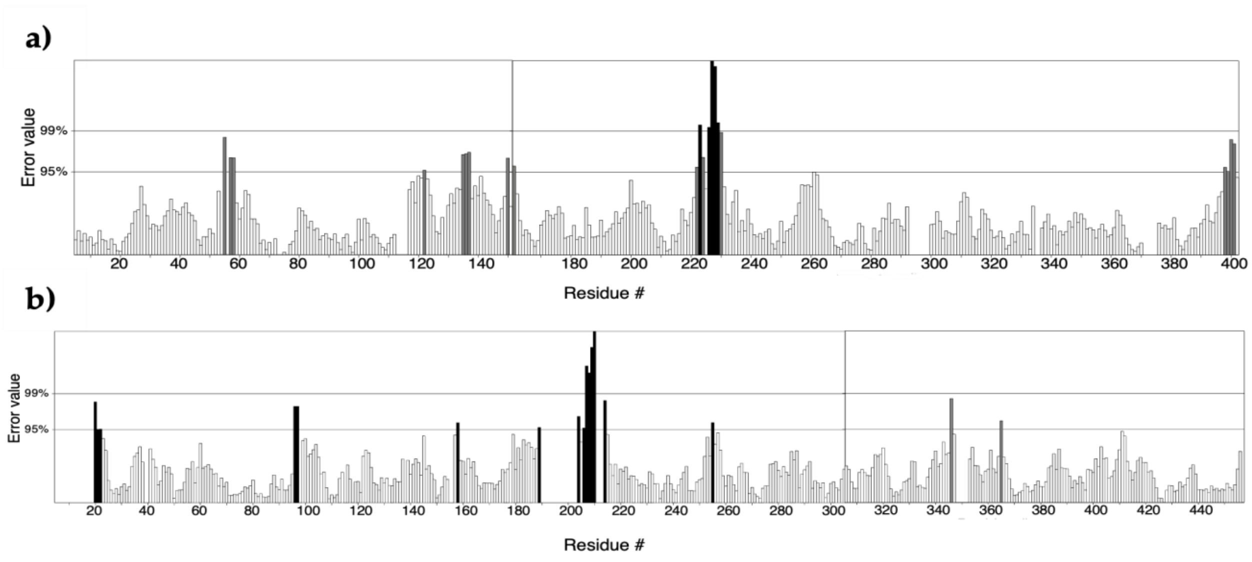

Figure S8. ERRAT plots for the APC structures. The error rates of the **(a)** HM and **(b)** AF structure are illustrated. White bars represent normal residues in the structure. Gray bars display residues with error rates between 90-95%, while black bars demonstrate misfolded regions with high error (>99%). The line in between represents the separation of chains.

a)

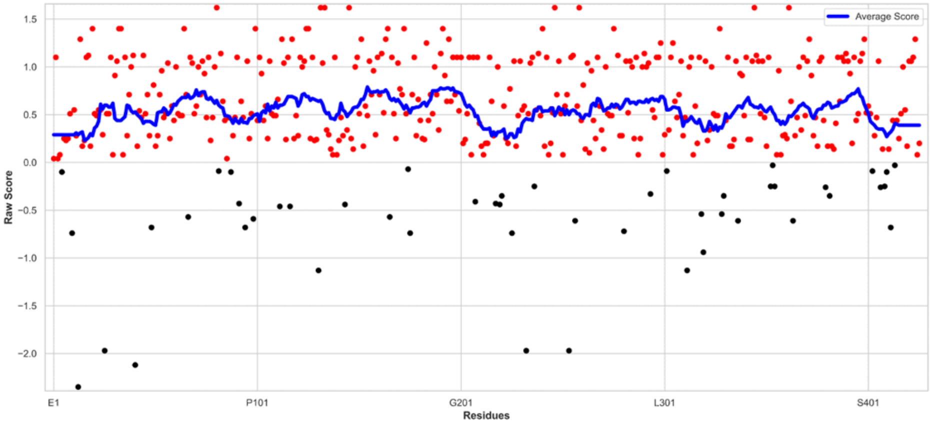

b)

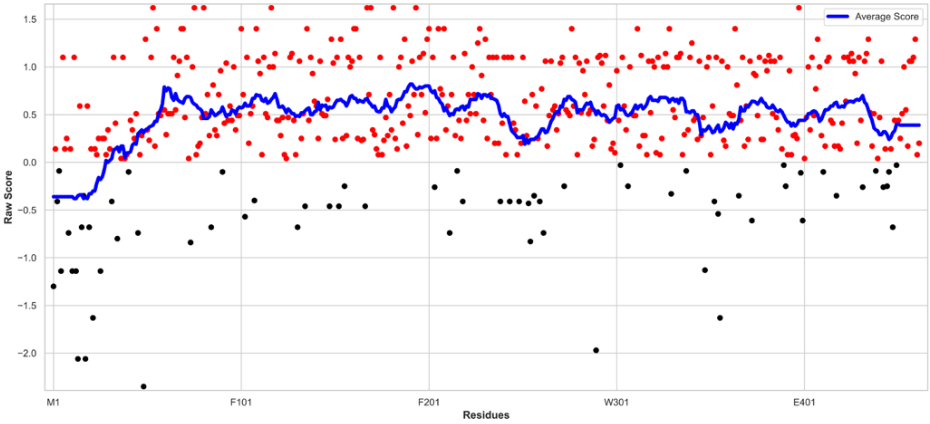

Figure S9. Verify3D plots for the Hemopexin structures**.** The average (blue line) and raw scores (red and black dots) of the residues for HM **(a)** and AF structure **(b)** were shown. Red dots indicate residues with a raw value above zero, while black dots point out the residues with negative values.

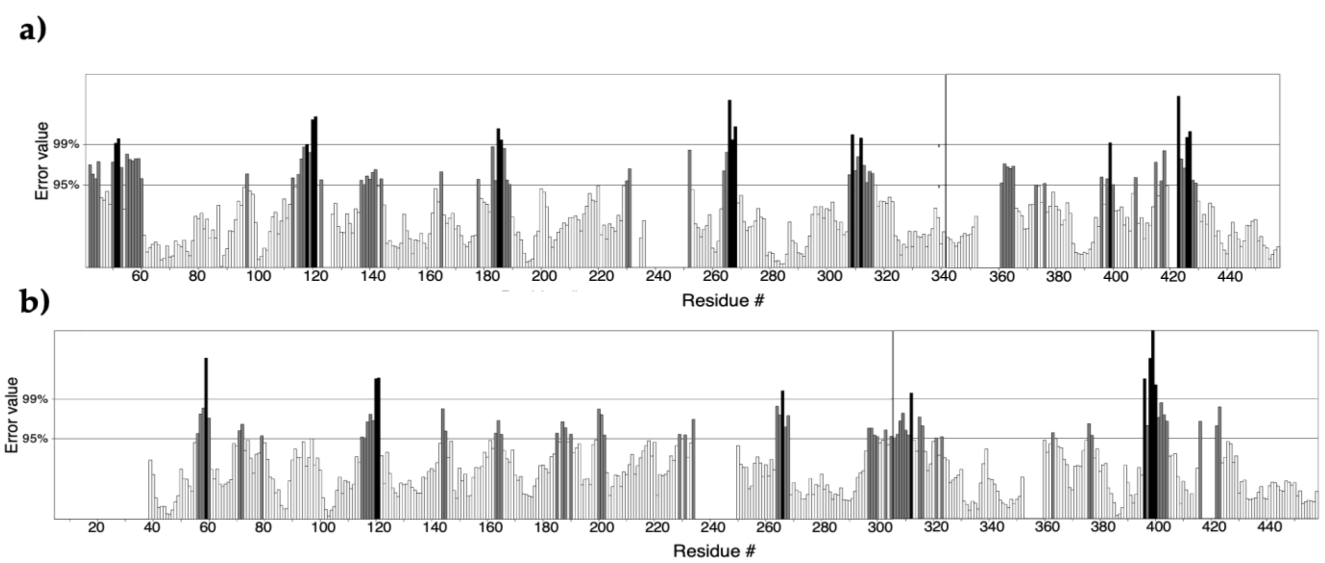

Figure S10. ERRAT plots for the Hemopexin structures**.** The error rates of the **(a)** HM and **(b)** AF structure is illustrated. White bars represent normal residues in the structure. Gray bars display residues with error rates between 90-95%, while black bars demonstrate misfolded regions with high error (>99%). The line in between represents the separation of chains.

a)

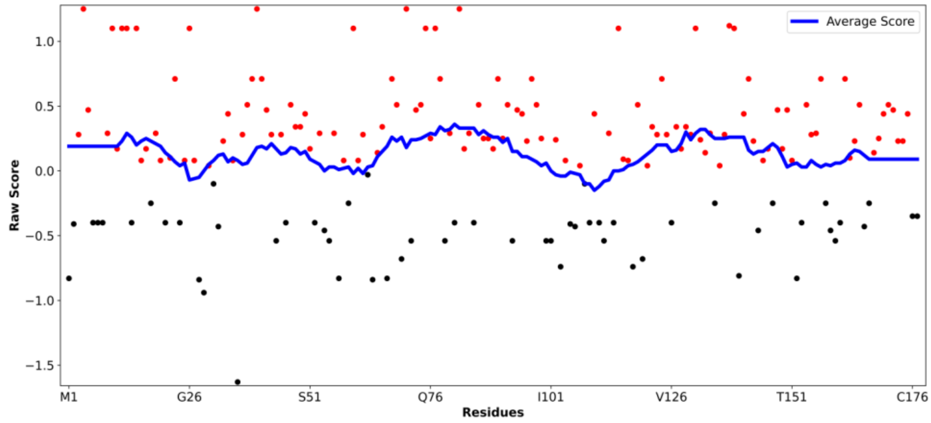

b)

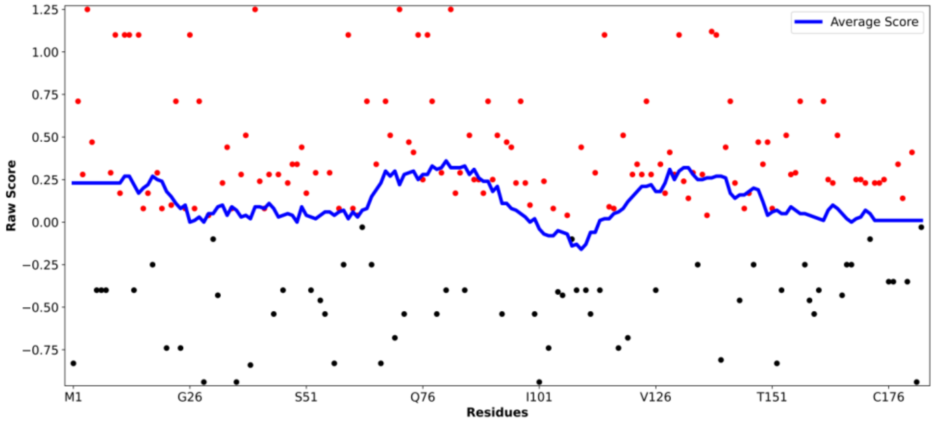

Figure S11. Verify3D plots for the Rap2 structures. The average (blue line) and raw scores (red and black dots) of the residues for HM **(a)** and AF structure **(b)** were shown. Red dots indicate residues with a raw value above zero, while black dots point out the residues with negative values.

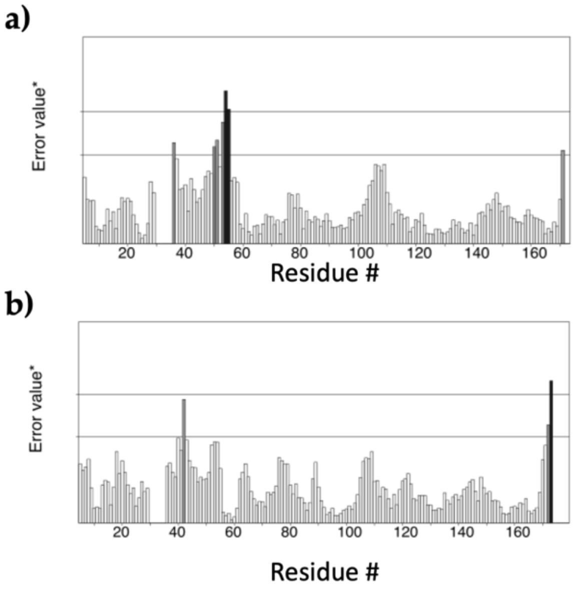

Figure S12. ERRAT plots for the Rap2 structures. The error rates of the **(a)** HM and **(b)** AF structure is illustrated. White bars represent normal residues in the structure. Gray bars display residues with error rates between 90-95%, while black bars demonstrate misfolded regions with high error (>99%). The line in between represents the separation of chains.

a)

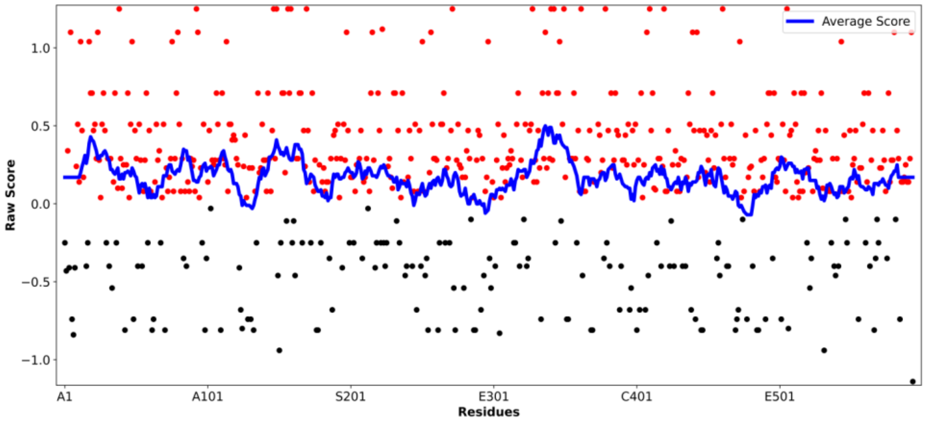

b)

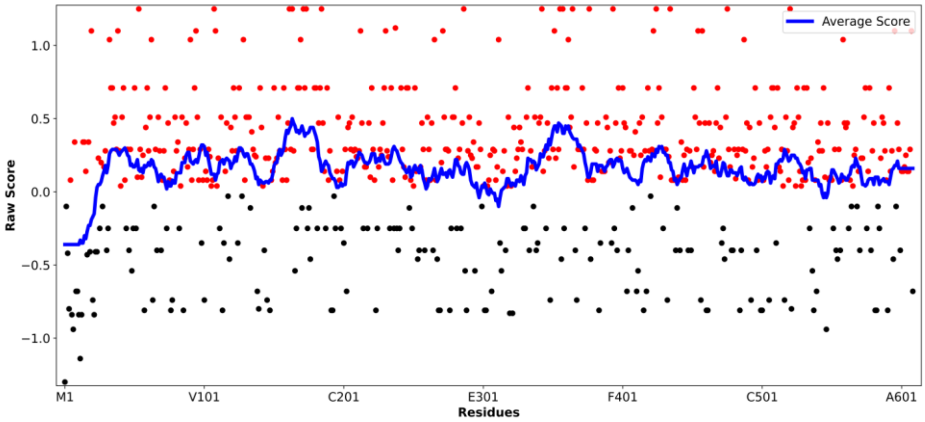

Figure S13. Verify3D plots for the HSA structures**.** The average (blue line) and raw scores (red and black dots) of the residues for HM **(a)** and AF structure **(b)** were shown. Red dots indicate residues with a raw value above zero, while black dots point out the residues with negative values.

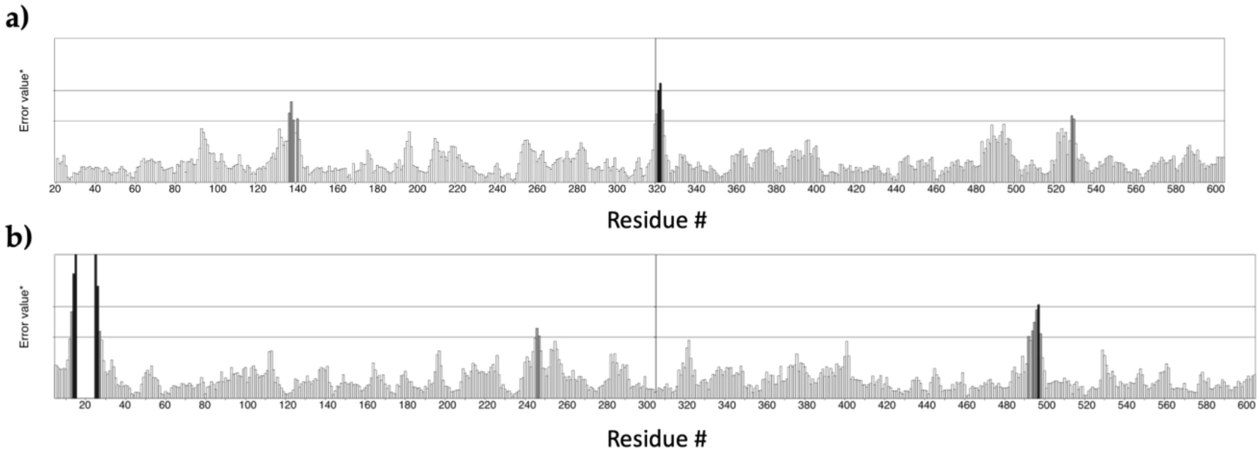

Figure S14. ERRAT plots for the HSA structures**.** The error rates of the **(a)** HM and **(b)** AF structure is illustrated. White bars represent normal residues in the structure. Gray bars display residues with error rates between 90-95%, while black bars demonstrate misfolded regions with high error (>99%). The line in between represents the separation of chains.

a)

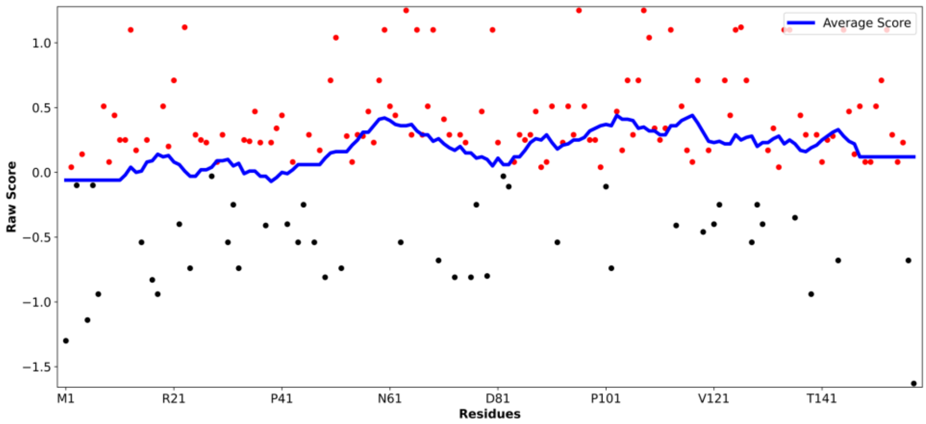

b)

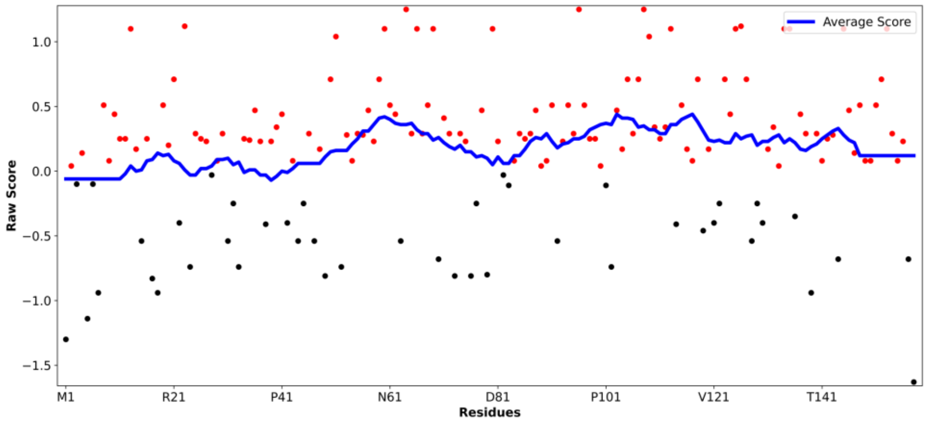

Figure S15. Verify3D plots for the IL 36*α* structures**.** The average (blue line) and raw scores (red and black dots) of the residues for HM **(a)** and AF structure **(b)** were shown. Red dots indicate residues with a raw value above zero, while black dots point out the residues with negative values.

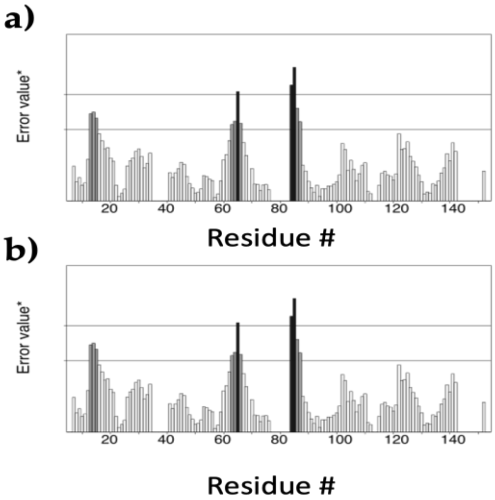

Figure S16. ERRAT plots for the IL 36α structures**.** The error rates of the **(a)** HM and **(b)** AF structure is illustrated. White bars represent normal residues in the structure. Gray bars display residues with error rates between 90-95%, while black bars demonstrate misfolded regions with high error (>99%). The line in between represents the separation of chains.

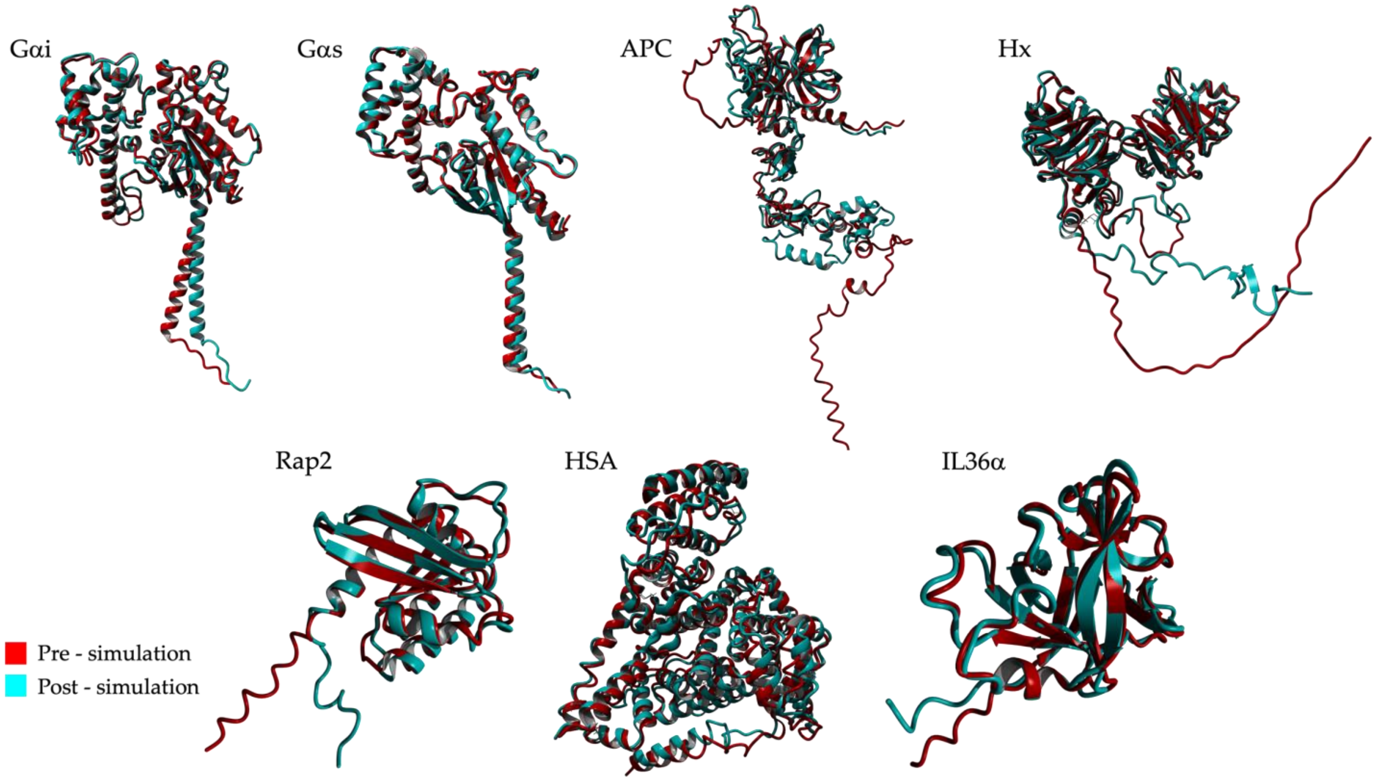

Figure S17. Structural alignments of the pre and post MD simulated structures from AF.

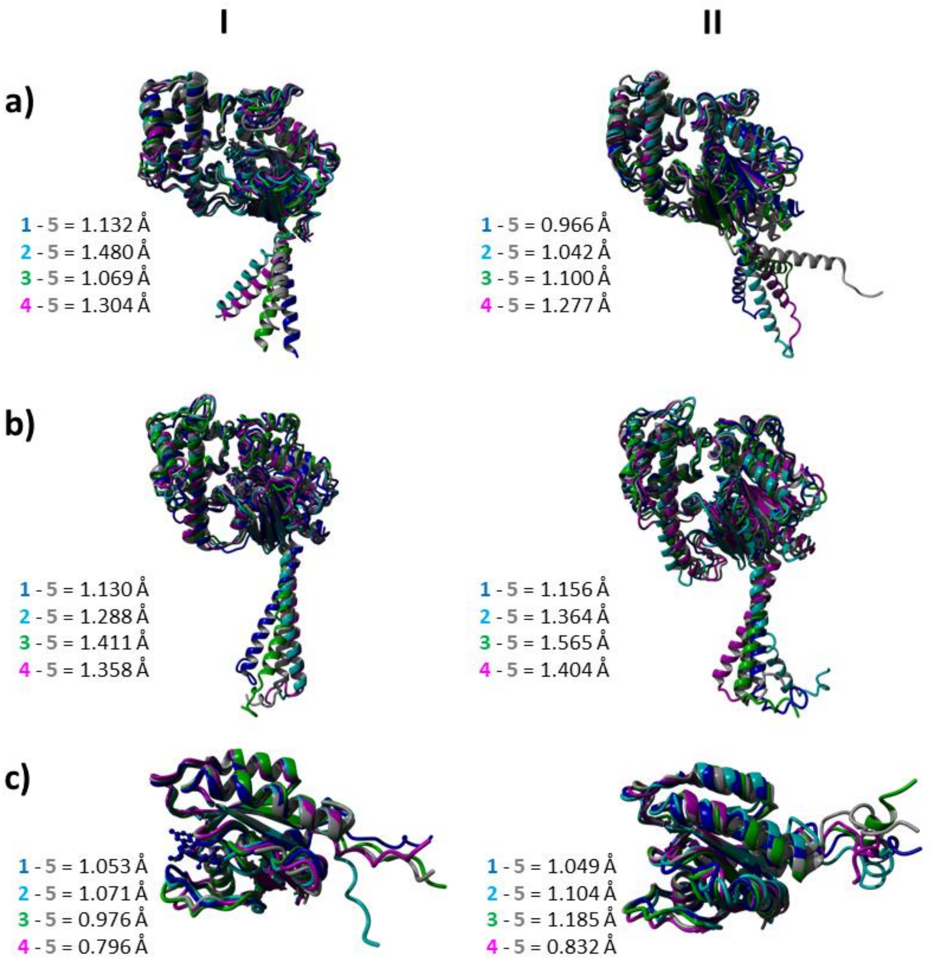

Figure S18. Selection of the representative post MD simulation structure for nucleotide binding proteins. Structural alignments of the selected structures of Gαi1, Gαs, and Rap2 are shown in panels **a**, **b**, and **c**, respectively. Column **I** shows the alignment of structures selected from those predicted by HM, while column **II** shows the alignment of structures predicted by AF. In the production phase of the MD simulation trajectory of each protein, four structures with extreme RMSD values plus the most recent structure were selected and structurally aligned. Each selected structure were indicated with a specific color code. The deviations between the chosen structures and the most recent structure, which was used in the analysis as the representative structure, were shown in the relevant panels. While no changes were observed in the overall conformation of the protein structures, deviations were observed to be due to the loop regions and the flexibility of the N-terminal helix.

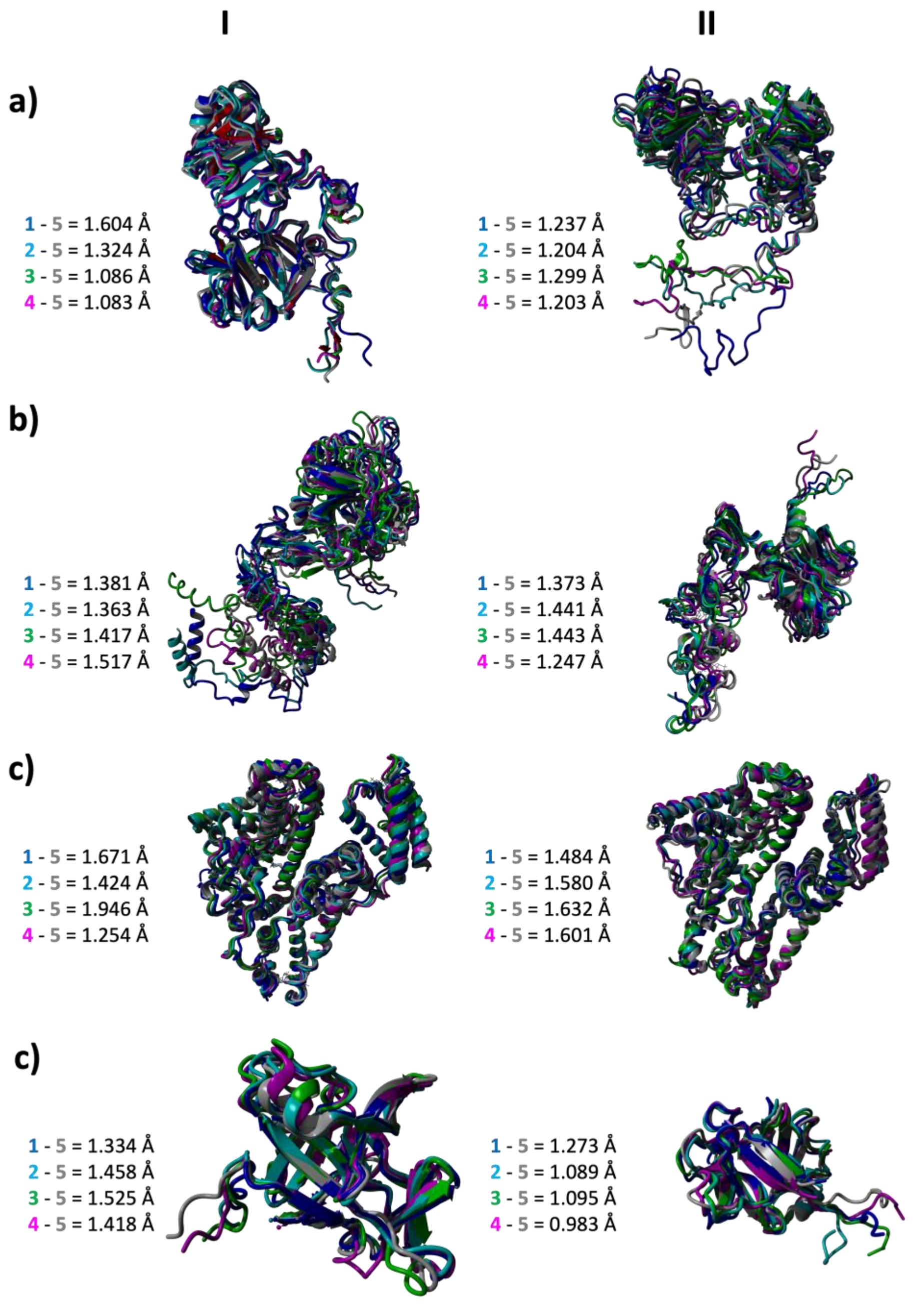

Figure S19. Selection of the representative post MD simulation structure for heme binding proteins. Structural alignments of the selected structures of APC, Hx, HSA, and IL 36α are shown in panels **a**, **b**, and **c**, respectively. Column **I** shows the alignment of structures selected from those predicted by HM, while column **II** shows the alignment of structures predicted by AF. In the production phase of the MD simulation trajectory of each protein, four structures with extreme RMSD values plus the most recent structure were selected and structurally aligned. Each selected structure were indicated with a specific color code. The deviations between the chosen structures and the most recent structure, which was used in the analysis as the representative structure, were shown in the relevant panels. While no changes were observed in the overall conformation of the protein structures, deviations were observed to be due to the loop regions and the flexibility of the N-terminal helix.

References

1. Goricanec, D.; Stehle, R.; Egloff, P.; Grigoriu, S.; Plückthun, A.; Wagner, G.; Hagn, F. Conformational Dynamics of a G-Protein α Subunit Is Tightly Regulated by Nucleotide Binding. Proc. Natl. Acad. Sci. USA **2016**, 113, E3629–E3638. https://doi.org/10.1073/pnas.1604125113.
2. Lambert, N.A.; Johnston, C.A.; Cappell, S.D.; Kuravi, S.; Kimple, A.J.; Willard, F.S.; Siderovski, D.P. Regulators of G-Protein Signaling Accelerate GPCR Signaling Kinetics and Govern Sensitivity Solely by Accelerating GTPase Activity. Proc. Natl. Acad. Sci. USA **2010**, 107, 7066–7071. https://doi.org/10.1073/pnas.0912934107.
3. Johnston, C.A.; Willard, F.S.; Jezyk, M.R.; Fredericks, Z.; Bodor, E.T.; Jones, M.B.; Blaesius, R.; Watts, V.J.; Harden, T.K.; Sondek, J.; et al. Structure of Gαi1 Bound to a GDP-Selective Peptide Provides Insight into Guanine Nucleotide Exchange. Structure **2005**, 13, 1069–1080. https://doi.org/10.1016/j.str.2005.04.007.
4. Liu, X.; Xu, X.; Hilger, D.; Aschauer, P.; Tiemann, J.K.S.; Du, Y.; Liu, H.; Hirata, K.; Sun, X.; Guixà-González, R.; et al. Structural Insights into the Process of GPCR-G Protein Complex Formation. *Cell* **2019**, *177*, 1243–1251.e12. https://doi.org/10.1016/j.cell.2019.04.021.
5. Dai, S.A.; Hu, Q.; Gao, R.; Blythe, E.E.; Touhara, K.K.; Peacock, H.; Zhang, Z.; von Zastrow, M.; Suga, H.; Shokat, K.M. State-Selective Modulation of Heterotrimeric Gαs Signaling with Macrocyclic Peptides. *Cell* **2022**, *185*, 3950–3965.e25. https://doi.org/10.1016/j.cell.2022.09.019.
6. Hu, Q.; Shokat, K.M. Disease-Causing Mutations in the G Protein Gαs Subvert the Roles of GDP and GTP. *Cell* **2018**, *173*, 1254–1264.e11. https://doi.org/10.1016/j.cell.2018.03.018.
7. Mather, T.; Oganessyan, V.; Hof, P.; Huber, R.; Foundling, S.; Esmon, C.; Bode, W. The 2.8 Å Crystal Structure of Gla-Domainless Activated Protein C. *EMBO J.* **1996**, *15*, 6822–6831. https://doi.org/10.1002/j.1460-2075.1996.tb01073.x.
8. Bajaj, S.P.; Schmidt, A.E.; Agah, S.; Bajaj, M.S.; Padmanabhan, K. High Resolution Structures of P-Aminobenzamidine- and Benzamidine-VIIa/ Soluble Tissue Factor: Unpredicted Conformation of the 192-193 Peptide Bond and Mapping of Ca2+, Mg2+, Na+, and Zn2+ Sites in Factor VIIa. *J. Biol. Chem.* **2006**, *281*, 24873–24888. https://doi.org/10.1074/jbc.M509971200.
9. Schmidt, A.E.; Padmanabhan, K.; Underwood, M.C.; Bode, W.; Mather, T.; Bajaj, S.P. Thermodynamic Linkage between the S1 Site, the Na+ Site, and the Ca2+ Site in the Protease Domain of Human Activated Protein C (APC): Sodium Ion in the APC Crystal Structure Is Coordinated to Four Carbonyl Groups from Two Separate Loops. *J. Biol. Chem.* **2002**, *277*, 28987–28995. https://doi.org/10.1074/jbc.M201892200.
10. Groebke Zbinden, K.; Banner, D.W.; Ackermann, J.; D'Arcy, A.; Kirchhofer, D.; Ji, Y.H.; Tschopp, T.B.; Wallbaum, S.; Weber, L. Design of Selective Phenylglycine Amide Tissue Factor/Factor VIIa Inhibitors. *Bioorganic Med. Chem. Lett.* **2005**, *15*, 817–822. https://doi.org/10.1016/j.bmcl.2004.10.092.
11. Shi, Y.; Zhang, J.; Shi, M.; O'Connor, S.P.; Bisaha, S.N.; Li, C.; Sitkoff, D.; Pudzianowski, A.T.; Chong, S.; Klei, H.E.; et al. Cyanoguanidine-Based Lactam Derivatives as a Novel Class of Orally Bioavailable Factor Xa Inhibitors. *Bioorganic Med. Chem. Lett.* **2009**, *19*, 4034–4041. https://doi.org/10.1016/j.bmcl.2009.06.014.
12. Paoli, M.; Anderson, B.F.; Baker, H.M.; Morgan, W.T.; Smith, A.; Baker, E.N. Crystal Structure of Hemopexin Reveals a Novel High-Affinity Heme Site Formed between Two β-Propeller Domains. *Nat. Struct. Biol.* **1999**, *6*, 926–931. https://doi.org/10.1038/13294.
13. Cherfils, J.; Ménétrey, J.; Le Bras, G.; Le Bras, G.; Janoueix-Lerosey, I.; De Gunzburg, J.; Garel, J.R.; Auzat, I. Crystal Structures of the Small G Protein Rap2A in Complex with Its Substrate GTP, with GDP and with GTPγS. *EMBO J.* **1997**, *16*, 5582–5591. https://doi.org/10.1093/emboj/16.18.5582.
14. Menetrey, J.; Cherfils, J. Structure of the Small G Protein Rap2 in a Non-Catalytic Complex with GTP. Proteins Struct. Funct. Genet. **1999**, 37, 465–473. https://doi.org/10.1002/(SICI)1097-0134(19991115)37:3<465::AID-PROT13>3.0.CO;2-O.
